# Lysosomal storage disease proteo/lipidomic profiling using nMOST links ferritinophagy with mitochondrial iron deficiencies in cells lacking NPC2

**DOI:** 10.1101/2024.03.26.586828

**Published:** 2024-03-27

**Authors:** Felix Kraus, Yuchen He, Sharan Swarup, Katherine A. Overmyer, Yizhi Jiang, Johann Brenner, Cristina Capitanio, Anna Bieber, Annie Jen, Nicole M. Nightingale, Benton J. Anderson, Chan Lee, Joao A. Paulo, Ian R. Smith, Jürgen M. Plitzko, Brenda A. Schulman, Florian Wilfling, Joshua J. Coon, J. Wade Harper

**Affiliations:** 1 Department of Cell Biology, Blavatnik Institute, Harvard Medical School, Boston, MA 02115, USA.; 2 Aligning Science Across Parkinson’s (ASAP) Collaborative Research Network, Chevy Chase, MD 20815, USA; 3 Department of Biomolecular Chemistry, University of Wisconsin–Madison, Madison, WI 53706, USA.; 4 Morgridge Institute for Research, Madison, WI 53715, USA.; 5 Department of Molecular Machines and Signaling, Max Planck Institute of Biochemistry, Martinsried, Germany.; 6 Mechanisms of Cellular Quality Control, Max Planck Institute of Biophysics, Frankfurt, Germany.; 7 CryoEM Technology, Max Planck Institute of Biochemistry, Munich, Germany.; 8 Department of Chemistry, University of Wisconsin–Madison, Madison, WI 53706, USA.; 9 equal contribution

**Keywords:** Lysosome storage disorders, proteomics, lipidomics, nano-LC, multi-omics, ferritinophagy, autophagy, OXPHOS, cryo-ET, mitochondrial dysfunction

## Abstract

Lysosomal storage diseases (LSDs) comprised ~50 monogenic diseases characterized by the accumulation of cellular material in lysosomes and associated defects in lysosomal function, but systematic molecular phenotyping is lacking. Here, we develop a nanoflow-based multi-omic single-shot technology (nMOST) workflow allowing simultaneously quantify HeLa cell proteomes and lipidomes from more than two dozen LSD mutants, revealing diverse molecular phenotypes. Defects in delivery of ferritin and its autophagic receptor NCOA4 to lysosomes (ferritinophagy) were pronounced in NPC2^−/−^ cells, which correlated with increased lyso-phosphatidylcholine species and multi-lamellar membrane structures visualized by cryo-electron-tomography. Ferritinophagy defects correlated with loss of mitochondrial cristae, MICOS-complex components, and electron transport chain complexes rich in iron-sulfur cluster proteins. Strikingly, mitochondrial defects were alleviated when iron was provided through the transferrin system. This resource reveals how defects in lysosomal function can impact mitochondrial homeostasis in trans and highlights nMOST as a discovery tool for illuminating molecular phenotypes across LSDs.

## INTRODUCTION

Lysosomes are the central organelle for degradative and recycling functions within eukaryotic cells, and digest cargo (e.g. macromolecules) delivered via various trafficking systems, including autophagy or endocytosis.^1,2^ Moreover, lysosomes are integral to lipid catabolism and nutrient sensing via the MTOR protein kinase. Genetic and clinical studies have linked lysosomal dysfunction to a wide variety of storage accumulation pathologies, called “lysosomal storage diseases”, or henceforth LSDs.^3^ More than 50 LSDs have been described and while individually rare, their combined prevalence is 1:5000 live births, while some population groups carry higher incidence rates. Not surprisingly due to the central role of lysosomes in cellular health, LSDs have been linked to various human diseases, including Niemann-Pick type C1/2 (*NPC1* and *NPC2*), Gaucher (*GBA1*), Pompe (*GAA*), Danon (*LAMP2*), and Neuronal Ceroid Lipofuscinoses (*GRN*). The majority of LSD genes encode catabolic enzymes (e.g. hydrolases) that function in the lysosomal lumen, although an important subset function as small molecule/ion transporters that maintain metabolic balance within the lysosome. Defects in these activities can result in a cascade of effects on downstream processes, leading to the accumulation of various types of materials: sphingolipids, mucopolysaccharides, glycoproteins, and lipofuscin.^3^ Additional storage material can also accumulate as a secondary response to the primary storage defect, including phospholipids, glycosphingolipids, and cholesterol. Several LSDs also lead to an imbalance in lipid catabolism and/or defects in sphingolipid metabolism (e.g. *CLN5* and *GRN*)^4,5^, raising the question of the extent to which dysregulation of lipid homeostasis underlies divergent LSDs.

Based on the diverse nature of storage material that can accumulate in LSDs and the central role of lysosomes as hubs for cellular health and signaling, LSDs present multisystem phenotypes and have been suggested to increase the risk of various diseases, including neurodegeneration.^3^ Large-scale genetic studies indicate association of a subset of LSDs with increased incidence of Parkinson’s Disease (PD), including *SMPD1*, *ASAH1*, ATP13A2, *CTSD* and *GBA1.*^6–9^ Approximately 10% of PD patients carry heterozygous mutations in *GBA1*, promoting earlier onset of the disease.^10,11^ Hence LSDs have been termed “canaries in the coalmine” for other neurological disorders and it is likely that additional links between lysosomal defects and neurodegenerative or other diseases are waiting to be discovered, creating a rich landscape for biological discovery that could inform new therapeutics.^12^

Here we systematically examine cells from a collection of engineered HeLa cell lines lacking or deficient in more than two dozen individual LSD genes using a newly developed nanoflow liquid chromatography (LC) Multi-Omic Single-shot Technology (nMOST) for simultaneous analysis of lipids and proteins. nMOST represents a second generation implementation of the microflow LC MOST method (mMOST)^13^ and integrates a multi-OMIC sample preparation strategy^14^ with intelligent lipidomics data acquisition.^4,15^ We show that this system provides deep proteome and lipidome coverage with high sensitivity across diverse sample types in a single mass spectrometry (MS) workflow. Application of nMOST across diverse LSD mutants revealed numerous allele-specific alterations in the proteome and lipidome of cells, facilitating LSD molecular phenotyping. Cross-correlation of proteome and lipidome led to the identification of an increase in autophagic factors and specific lipids (particularly LysoPC, phosphatidylcholine and short chain cholesterol ester lipids) in cells from Niemann-Pick disease type C1/2 (*NPC1*, *NPC2*) and *LIPA* mutant cells. The lysosome-resident multipass transmembrane protein NPC1 receives free (unesterified) cholesterol from the lumenal carrier protein NPC2 and transports cholesterol and sphingosine lipids to the cytosol for subsequent delivery to other cellular membranes^16^, while LIPA cleaves cholesterol esters (CE) in the lysosomal lumen to generate cholesterol for transport by NPC1/NPC2. NPC1 loss of function has previously been linked with defects in autophagy^17–19^, albeit through incompletely understood mechanisms.

Using 3D-Structured Illumination Microscopy (3D-SIM), we found that cells lacking *NPC2* accumulate autophagic cargo and receptors adjacent to or co-incident with the lysosomal limiting membrane in cholesterol-laden lysosomes that harbour highly ordered multi-lamellar membranes as visualized by cryo-electron tomography (cryo-ET). One such autophagic cargo was Ferritin, which sequesters iron and is delivered to the lysosome via the ferritinophagy receptor NCOA4.^20,21^ Ferritin degradation within the lysosomal lumen allows release of iron to the cytosol and other organelles for assembly into diverse iron-sulfur (Fe-S) cluster proteins. Total proteome analysis of *NPC2*^−/−^ cells revealed reduced levels of components within the mitochondrial oxidative phosphorylation (OXPHOS) machinery, which relies extensively on Fe-S cluster proteins. Reduced levels of OXPHOS proteins correlated with a reduction in the frequency of mitochondrial cristae and reduced MICOS (mitochondrial contact site and cristae organizing system) and MIB (mitochondrial inner membrane space bridging complex) components. Strikingly, defects in OXPHOS abundance, as well as cristae number and the abundance of MICOS-MIB components in *NPC2*^−/−^ cells, are largely rescued upon delivery of iron to cells via the lysosome-independent transferrin system, indicating that an inability to release iron by ferritinophagy underlies defects in mitochondrial function in *NPC2*^−/−^ cells. This LSD proteomic and lipidomic landscape provides a resource for deep molecular phenotyping of lysosomal and cellular functions with links to diverse diseases, and reveals the consequences of lysosomal dysfunction on mitochondrial functions requiring iron.

## RESULTS

### Robust proteomic and lipidomic analysis using nMOST

**Figure 1.**
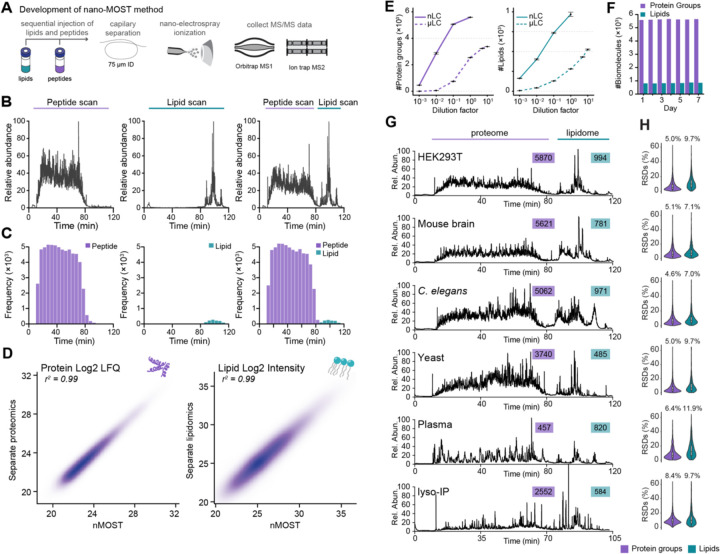
Development and benchmarking of nMOST for simultaneous proteomics and lipidomics analysis. **(A)** Schematic of the nMOST method, which allows simultaneous proteome and lipidome analysis by LC-MS. Lipid and protein extracts isolated from the same cell sources are sequentially injected onto LC prior to elution with an organic gradient and MS analysis (see METHODS). **(B)** Chromatograms showing HEK293 cell peptide and lipid elution features during a 120 min gradient examining (left panel) total protein extract, (middle panel) total lipid extract, and (right panel) sequentially loaded protein and lipid extracts and nMOST analysis. The vast majority of peptides elute before 80 min while the majority of lipids elute between 80 and 120 min. **(C)** Peptide and lipid identifications from the corresponding LC-MS run in panel B. **(D)** Correlation of proteins (left panel) and lipids (right panel) identified by separate LC-MS (y-axis) versus nMOST (x-axis). r2 values are >0.99. **(E)** Number of protein groups and lipid groups identified by nMOST versus mMOST methods. nMOST routinely out-performed mMOST for both proteins (left panel) and lipids (right panel). **(F)** Performance was comparable for both proteins and lipids when measured daily over a 7-day acquisition period. **(G)** nMOST allows simultaneous analysis of proteins and lipids from HEK293 cells, mouse brain extracts, *C. elegans* extracts, budding yeast extracts, human plasma, and lysosomes from HeLa cells isolated by Lyso-IP. **(H)** RSD values for the data in panel G.

We previously reported μMOST as a robust and high-throughput method to acquire proteomics and lipidomics data simultaneously.^13^ However, the sensitivity of microflow was limited, which prompted the development of an analogous nanoflow method with substantially increased sensitivity (Figure 1A). nMOST takes advantage of the fact that the vast majority of lipid species elute from reverse phase columns well after the vast majority of peptides, as indicated when peptide and lipid extracts from HEK293 cells are analyzed separately with the same mobile phase gradient (Figure 1B,C, left and middle panels). We found, however, that sequential loading of lipid and peptide extracts followed by LC-MS provided virtually identical performance (Figure B,C, right panels), with correlation coefficients (*r*) for both protein label free quantification (LFQ) and lipid intensity > 0.99 (Figure 1D, Figure S1A). Thus, the presence of peptides on the immobile phase did not affect lipid detection or quantification and vice versa. Consistent with added sensitivity of the nanoflow approach, nMOST delivered >2-fold more protein (5593 vs 2540) or >3-fold more lipid (967 vs 281) identifications as compared with μMOST when analyzing 1 μg of HEK293 cell peptides or 0.03% lipid mass in a single LC-MS run (Figure 1E). Moreover, the method was found to be robust, with a similar number of biomolecules quantified over an extended period of data collection, with a median relative standard deviation (RSD) of 5.0% for proteins and 13.2% for lipids (Figure 1F, Figure S1B–D and see below).

To demonstrate versatility, we benchmarked nMOST performance across multiple species (HEK293 cells, mouse brain, *C. elegans*, budding yeast) and sample types (cell extracts, plasma, purified lysosomes) (Figure 1G). We observed the expected complexity of proteomes across the various samples, and routinely detected thousands of proteins and ~500–1000 lipid species (Figure 1G), with RSDs of 4.6–8.4% for proteins and 7.0–11.9% for lipids (Figure 1H). The consistent identifications and stable quantifications over extended periods of analysis time highlight the robustness of the method and reinforce its potential for reproducible and high-quality data acquisition to elucidate complex relationships between proteomes and lipidomes.

### A Tool-kit for Systematic Analysis of LSD Genes

To investigate the molecular landscape of LSDs, we used CRISPR/Cas9 to attempt targeting of 52 LSD genes in HeLa^TMEM192-HA^ cells (Figure S1E–G, Table S1).^22,23^ Through a combination of DNA sequencing or proteomics of clonal, edited cells, we validated a total of 37 mutants across multiple functional classes of LSDs: 23 homozygous deletions, 5 heterozygous deletions, and 10 mutants containing one or more alleles with an in-frame deletion (Figure S1E–G; Table S1). In the majority of cases for heterozygous and in-frame deletion clones, the levels of target proteins detected by either proteomics, when detected, indicated substantially reduced protein levels (Figure S1E–G; see Table S1 for detailed genotyping results of each clone). We excluded heterozygous deletions in the subsequent analysis, leaving 33 LSD mutant cell lines which serve as a resource for phenotypic analysis of a broad range of LSD genes.

### Molecular fingerprinting of LSDs using nMOST

**Figure 2.**
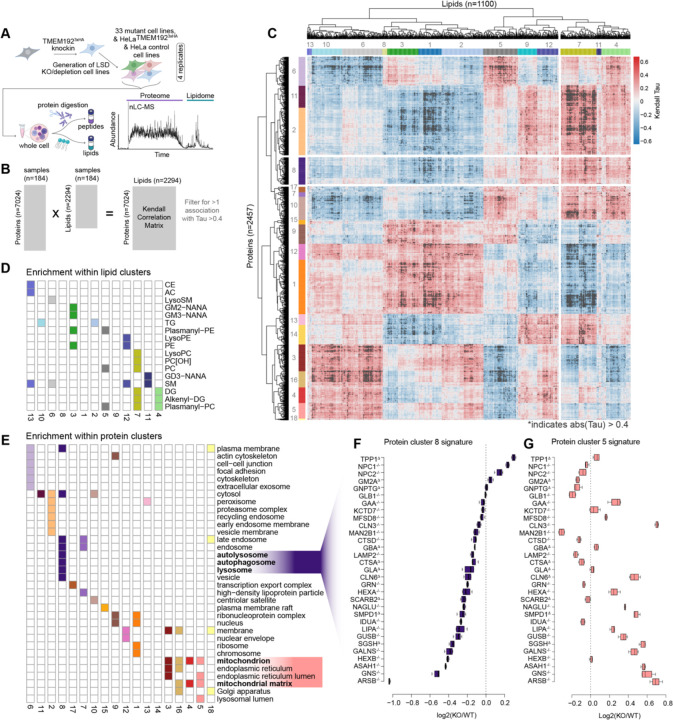
Landscape of total proteomes and lipidomes from LSD mutant cells using nMOST. **(A)** Schematic describing the method for analysis of total cell extracts across 33 LSD mutants. Protein and lipid extracts were isolated from the samples in quadruplicate, and then sequentially injected for analysis by LC-MS over a 120 min gradient. **(B,C)** Panel B is a schematic depicting the method used for lipid/protein cross-correlation analysis employing a Kendall rank correlation (filtered for >1 association with Tau >0.4). Panel C shows a heatmap for Tau values. Clusters for proteins and lipids are shown. **(D)** Schematic showing the enrichment of specific lipids within individual lipid clusters. **(E)** Schematic showing the subset of GO term Cellular Compartment enriched within individual protein clusters. **(F)** Summed protein cluster 8 signature (sum abundance of all proteins within cluster 8 (enriched for autophagy terms) across the LSD mutant cells plotted as log_2_FC (KO/WT). **(G)** Signature of protein cluster 5 (sum protein abundance relative to WT) across the LSD mutant cells.

We applied the nMOST method to total cell extracts using quadruplicate independent cultures (Figure 2A). This was accomplished by running the samples across 15 sets, where each set contain multiple HeLa^TMEM192-HA^ or Control parental samples and were flanked by instrument quality control (QC) runs, ensuring stable performance. In total, 318 whole cell extract samples were subjected to nMOST, representing 4 weeks of cumulative continuous data collection, with little change in method performance, as indicated by the log_2_ Quant values for proteins and lipids. Additionally, 45 QC samples spread throughout the data collection period demonstrated high performance reproducibility (Figure S1G). In total, over 7000 proteins and 2000 lipids were routinely identified and quantified in whole cell extracts (Table S2,S3).

To identify molecular fingerprints across the LSD mutant cells, we performed a cross-ome correlation analysis using a Kendall rank approach (Figure 2B), resulting in 1100 lipids and 2457 proteins with at least two correlations |Tau| >0.4. Hierarchical clustering of the correlation matrix revealed 13 lipid and 18 protein clusters (Figure 2C, S2A) with significant enrichment of either lipid-classes or subcellular compartments across the proteo-lipidomic landscape for 31 LSD mutant cell lines (Figure 2D,E). Given the importance of autophagy in cellular homeostasis and neurodegeneration, we focused on protein cluster 8, encompassing lysosome, autolysosome and autophagosome terms and correlated significantly with phosphocholines (PC), plasmanyl-PCs, diacylglycerols (DGs), alkenyl-DGs, and gangliosides (Figure 2D,). The summed cluster 8 signature plotted as log_2_(KO/Control) indicated that *NPC1*^−/−^ and *NPC2*^−/−^ were among the strongest candidates for proteins within the lysosome cluster, cluster 8 (Figure 2F, Table S3). NPC1 and NPC2 proteins are involved in the export of cholesterol from the lysosome while LIPA is a lumenal lysosomal enzyme that produces cholesterol from cholesterol esters (CEs). We found that *LIPA*^−/−^ was enriched in clusters 4 and 5, which are distinguished by increased levels of mitochondrial proteins with little alteration in autophagy proteins (Figure 2G).

To further deconvolve which organelles and processes were most affected by altered cholesterol efflux from lysosomes, we created a curated sublist of organelle proteins (1784 IDs), encompassing annotated proteins for mitochondria, lysosome, endosome, Golgi, ER, proteasome and as well as autophagy and iron homeostasis and performed kmeans clustering (Figure S2C,D; D contains average abundance of indicated annotation group). *NPC1*^−/−^ and *NPC2*^−/−^ clustered closely together (Group 2) while *LIPA*^−/−^ was located in Group 5, consistent with differential effect on organelle proteomes. We focused on three clusters of interest: While proteins belonging to cluster 3 (GO: [regulation of] mitochondrial RNA catabolic process) were increased across all three genotypes, cluster 4 (GO: [macro-] autophagy & vacuole organization) was elevated in Group 2 mutants containing *NPC1*^−/−^ and *NPC2*^−/−^, but not in *LIPA*^−/−^ in Group 5, while cluster 5 (GO: ATP synthesis & aerobic electron transport chain) was increased in Group 5 but not Group 2. Thus, the signatures observed in Figure 2B–E for *NPC* mutants and *LIPA*^−/−^ may reflect changes in organelle homeostasis, especially mitochondria and autophagy. Moreover, PCs, LysoPC and Plamanyl-PCs found in lipid cluster 7 was highly correlated with autophagy regulators (Figure S2C–E), as discussed further below. Collectively, these data raised the question of how accumulation of cholesterol in the lysosome might be linked with alterations in both autophagy and mitochondrial protein abundance.

### NPC1 and NPC2 mutants accumulate juxta-lysosomal autophagy receptors

**Figure 3.**
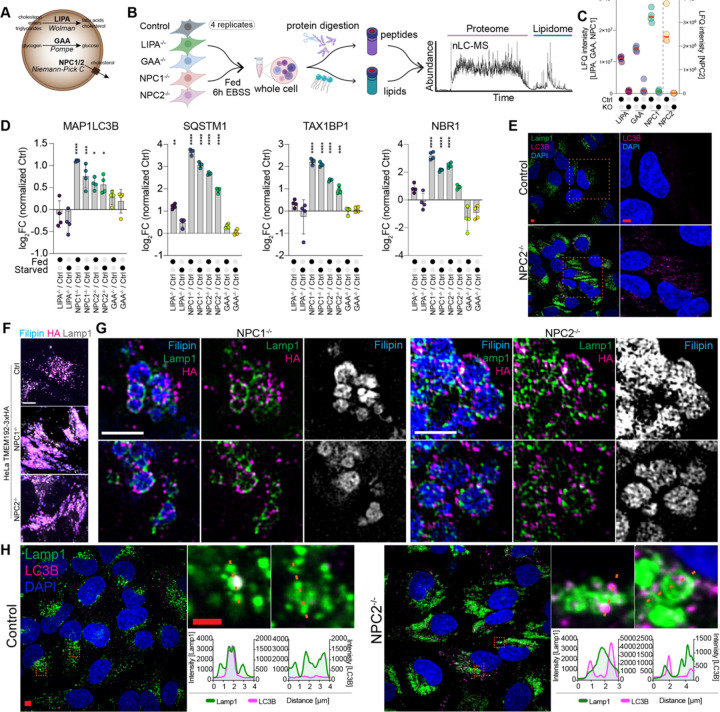
Juxta-lysosomal accumulation of autophagy receptors in *NPC1*^−/−^ and *NPC2*^−/−^ cells. **(A,B)** Application of nMOST for analysis of Control, *LIPA*^−/−^, *GAA*^−/−^, *NPC1*^−/−^ and *NPC2*^−/−^ cells (4KO cells). The general functions of the four proteins within the lysosome is shown in panel A and the nMOST workflow is shown in panel B. **(C)** LFQ of LIPA, GAA, NPC1 and NPC2 in Control and mutant cells based on nMOST data. Data based on quadruplicate replicate nMOST measurements. **(D)** Log_2_FC relative to Control cells for the indicated autophagy receptors for 4KO cells. MAPLC3B: p(****) <0.0001, p(***) = 0.0001, p(*)=0.0129 & 0.0157. SQSTM1: p(****) <0.0001, p(**) = 0.0047. TAX1BP1: p(****) <0.0001, p(***) = 0.0001. NBR1: p(****) <0.0001. Data based on quadruplicate replicate nMOST measurements, ordinary one-way ANOVA with multiple comparisons, alpha = 0.05. Error bars depict S.D. **(E)** Immunostaining of Control and *NPC2*^−/−^ cells with α-LAMP1 and α-LC3B. Nuclei were stained with DAPI. Scale bars = 5 μm. **(F)** The indicated cells were stained with Filipin to stain cholesterol-rich lysosomes and immunostained with α-LAMP1 and α-HA to detect TMEM192HA in lysosomes, followed by imaging with confocal microscopy. Scale bars = 10 μm. **(G)** Cells from panel F were imaged using 3D-SIM. Scale bar = 2 μm. **(H)** Confocal images of immunostaining of Control and *NPC2*^−/−^ cells with α-LAMP1, α-LC3B and nuclei stained with DAPI. Line trace plots across individual Lamp1-positive lysosomes and the corresponding LC3B intensities. Scale bars = 5 μm and 2 μm (insets).

Among the summed cross correlations, *NPC1*^−/−^ and *NPC2*^−/−^ mutants showed high correlation between alkenyl-DGs, Plasmanyl-PCs, GD3-NANA and LysoPC lipid species and autophagy-related proteins, including ATG8 proteins GABARAPL1/2, ubiquitin-binding cargo receptors TAX1BP1 and SQSTM1, the ferritinophagy receptor NCOA4 (Figure S2B,C). Moreover, organelle-annotated proteins, including mitochondria and OXHOPHS components, clustered in *NPC* mutants, suggesting a potential link between them (Cluster 8 in Figure S2E). Previous studies reported an increase in the abundance of an overlapping set of autophagy receptors in lysosomes isolated from *NPC1*^−/−^ cells.^17,18,24^ To both validate and further examine this correlation, we selected *NPC1*^−/−^, *NPC2*^−/−^, *LIPA*^−/−^ cells together with *GAA*^−/−^ mutant and parental HeLa^TMEM192-HA^ cells as controls for systematic analysis (Figure 3A). Given the important role of lysosomes in sensing and responding to nutrient stress via MTOR to induce autophagy, we performed nMOST analysis of this “4KO” cohort in quadruplicate under both full media (= Fed) and nutrient stress (EBSS, 6h) conditions (Figure 3B, Table S4). The absence of the deletion target was verified by label-free quantification of nMOST data (Figure 3C) and PCA analysis of nMOST data revealed high sample and treatment reproducibility (Figure S3A,B), pointing to the robustness of the nMOST method. Consistent with previous studies examining lysosomes in *NPC1*^−/−^ cells^17^, we observed an increase in the abundance of Ub-binding receptors SQSTM1, TAX1BP1, and NBR1, as well as LC3B (MAP1LC3B), in mutants under both Fed and EBSS-treated conditions, particularly in *NPC1*^−/−^ and *NPC2*^−/−^ cells (Figure 3D). Previous work using confocal microscopy concluded that LC3B accumulated within lysosomal lumen in *NPC1*^−/−^ cells, and proposed defects in lysosomal degradation being responsible for receptor accumulation, yet substantial LC3B signal appeared to be juxta-lysosomal rather than lumenal in *NPC1*^−/−^ cells, unlike Control cells.^17^ We also observed increased LC3B and SQSTM1 puncta in *NPC1*^−/−^ and *NPC2*^−/−^ cells; however distinguishing luminal from a juxta-lysosomal localization of SQSTM1 or LC3B was difficult to ascertain (Figure 3E, S3C).

To examine autophagic cargo localization in greater detail, we first used the cholesterol-binding fluorescent molecule Filipin to mark cholesterol-rich lysosomes^25,26^ and LAMP1 or TMEM192^HA^ to mark lysosomal membranes. As expected, *NPC1*^−/−^ and *NPC2*^−/−^ cells, but not Control, *LIPA*^−/−^ or *GAA*^−/−^, accumulated abundant Filipin staining by confocal imaging (Figure S3D), and lysosomes in *NPC1*^−/−^ and *NPC2*^−/−^ cells displayed elevated pH (5.9 and 6.5, respectively, compared with a pH of 5.5 in control cells) in line with previous reports^27^ (Figure S3E,F). Confocal microscopy showed extensive overlap between lysosomal membrane proteins and Filipin signal, however, due to the enlarged lysosomal size in *NPC1*^−/−^ and *NPC2*^−/−^, LAMP1 and TMEM192^HA^ rings around the cholesterol-rich lysosomal core became visible (Figure 3F). To further address the spatial organization of lysosomes, we next employed volumetric 3D-SIM, which revealed that the numerous enlarged Filipin-positive lysosomes (typically >0.5 microns) present in *NPC1*^−/−^ and *NPC2*^−/−^ cells were studded with distinctive LAMP1 and TMEM192^HA^ puncta that were typically non-overlapping in individual z-slices (Figure 3G). Linescan analysis on confocal images across lysosomes indicated that LAMP1 signal coated the perimeter of Filipin signal, consistent with LAMP1 localization in the lysosomal limiting membrane (Figure S3G). As Filipin staining was, unfortunately, incompatible with α-LC3B staining fixation conditions, we employed LAMP1 staining to mark the lysosomal limiting membrane and examined the relative location of LC3B puncta in single z-sections of Control and *NPC2*^−/−^ cells (Figure 3H). Lysosome expansion in *NPC2*^−/−^ cells frequently allowed identification of LAMP1-positive rings, and we observed LC3B puncta either coincident with LAMP1-positive rings or outside these LAMP1-positive structures, as also revealed by linescan analysis (Figure 3H). In contrast, while Control cells had much smaller lysosomes and far fewer LC3-positive puncta under fed conditions; these tended to be co-incident with LAMP1 signal (Figure 3H). These results are consistent with an inability to successfully deliver autophagic cargo to the lysosomal lumen in *NPC2*^−/−^ cells. We wondered if LC3B’s localization near or coincident with the outer lysosomal membrane is related to the cholesterol accumulation observed in NPC mutants from an ultrastructural perspective, meaning that lysosomes in NPC mutants display diminishing autophagic capacity not only due to the increase in pH, but also due to the structural rearrangements that accompany cholesterol deposition.

### Visualization of organelle ultrastructure in *NPC2*^−/−^ cells by cryo-ET

**Figure 4:**
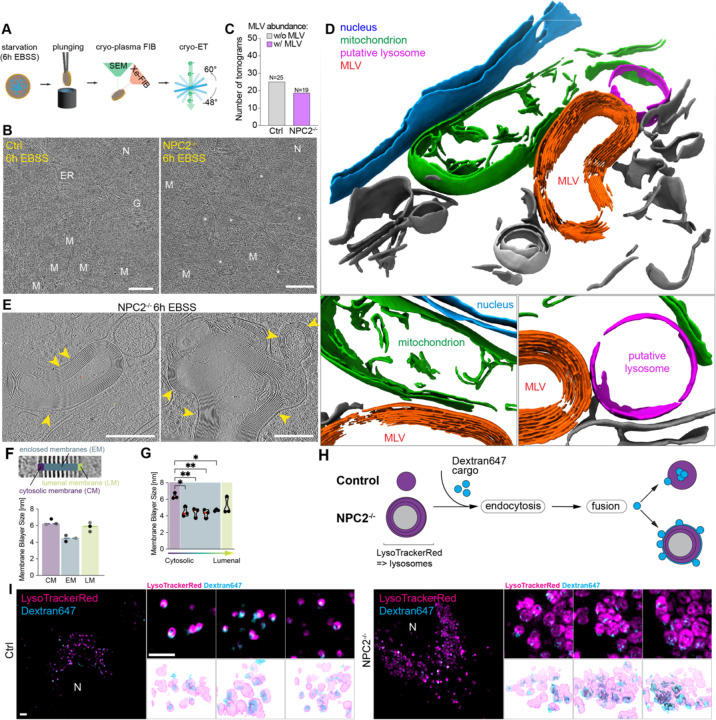
Visualization of multi-lamellar membranes in *NPC2*^−/−^ lysosomes by cryo-ET. **(A)** Schematic of the plasma-FIB and cryo-ET workflow. **(B)** Example lamella overviews of Control and *NPC2*^−/−^ cells under 6h EBSS nutrient starvation conditions. Scale bar = 500 nm. **(C)** Quantification of MLV-containing tomograms from Control and *NPC2*^−/−^ cells. Total number of tomograms analyzed is stated above the bar charts. (**D**) 3D-renderings of a segmented *NPC2*^−/−^ tomogram. Zoom-ins highlighting close proximity between MLV (orange) with mitochondria (green) and a putative lysosome (pink) are shown beneath. **(E)** Example tomogram slice of multi-lamellar vesicles in *NPC2*^−/−^ cells. Scale bar = 200 nm. **(F,G)** Quantification of membrane bilayer size (F) and distance between membrane leaflets (G) across three tomograms for the cytosolic membrane (CM), the enclosed membranes (EM), and the luminal membrane (LM). Quantification of the spacing between individual membranes: CM to first EM (left), between Ems (middle), and EM to LM (right). p(*) = 0.011, 0.21; p(**) = 0.0086, 0.0052. Data based on triplicate experiments (lamellae), ordinary one-way ANOVA with multiple comparisons, alpha = 0.05. Error bars depict S.D. **(H)** Schematic showing endocytosis of dextran and its ultimate incorporation into the lysosome in Control and *NPC2*^−/−^ cells. In Control cells, dextran endocytosis successfully delivers dextran to the lysosomal lumen via vesicle fusion. In *NPC2*^−/−^ cells with multi-lamellar membranes, successful fusion and delivery of dextran is reduced and successful fusion events result in dextran present in the limited lumenal space between the limiting lysosomal membrane and the first internal membrane. **(I)** Control or *NPC2*^−/−^ cells were treated with dextran conjugated with alexa-647 dye and imaged by structured illumination microscopy. Images derived from 3D-SIM reconstructions are shown. Scale bar = 2 μm. Abbreviations: ER = Endoplasmic reticulum; MLV = Multi-lamellar vesicle.

Enlarged cholesterol-laden lysosomal structures in *NPC2*^−/−^ cells prompted us to ask whether alterations in lysosome structure might underlie the accumulation of autophagic receptors. We therefore sought to study lysosomal ultrastructure at higher resolution than is possible by light microscopy. Transmission electron microscopy (TEM) using formaldehyde-based cell fixation and negative staining revealed numerous abnormal vesicular structures in *NPC1*^−/−^ and *NPC2*^−/−^ cells, which contained densely stained membrane structures reminiscent of intralumenal vesicles (Figure S3H). Such structures were rare in Control cells. To examine the morphology of these structures *in situ*, we made use of semi-automated cryo-plasma FIB (cryo-PFIB) milling paired with cryo-ET. (Figure 4A, Figure S4A, see METHODS for more details, ^28^). Matching observation by negative-stain TEM, *NPC2*^−/−^, but not Control cells, treated with EBSS harboured numerous multi-lamellar vesicles (MLVs). These were already frequently seen on the low-magnification TEM-overviews (Figure 4B, white stars). In contrast, similar structures were not detectable in the lamella overviews of control cells. Based on the size and cellular localization of these MLVs, we hypothesized that these structures correspond to the aberrant, cholesterol-filled lysosomes characterized by fluorescent microscopy (Figure 3F,G, Figure S3G). Analysis of the corresponding tomograms (25 for Control and 19 for *NPC2*^−/−^ cells, respectively) showed that the majority of MLVs in *NPC2*^−/−^ cells were structurally aberrant, containing as many as 17 highly organized membranes surrounding a lumen. In contrast, such structures were absent in tomograms from EBSS-treated Control cells (Figure 4C). Tomogram segmentations highlight the membrane organization of *NPC2*^−/−^ MLVs in cross-section (Figure 4D) and their close proximity to mitochondria and a putatively intact lysosome nearby. Interestingly, the intermembrane distance inside MLVs was highly regular (2.6 +/− 0.2 nm between membranes at half maximum), whereas the distance of the outer cytosolic membrane to the first enclosed membrane was highly variable across MLVs (Figure 4E yellow arrows, Figure S4B–D). For the cytosolic membrane, both leaflets were clearly distinguishable with full width at half maximum (FWHM) of 6.4 ± 0.4 nm. (Figure 4F,G). However, the interleaflet space of the enclosed membranes and the luminal membrane could not be resolved at the magnification used and showed an FWHM of 4.5 ± 0.4 nm and 5.8 ± 0.5 nm, respectively. (Figure 4F,G, S4B–C).

Accumulation of MLVs suggested potential alterations in lipids abundance, possibly reflective of increased cholesterol within the lysosome. We therefore employed nMOST data to globally examine lipid alterations in NPC mutant cells. Cholesterol ester abundance was generally elevated in whole-cell lipidomics in *NPC2*^−/−^ cells, as indicated by a skew of CE species towards the upper end of the ranked abundance after starvation relative to Control cells (Figure S4E). This was particularly evident for CE species with chain lengths shorter than 20 carbons (Figure S4E, right panels). The selective abundance increase in <20-chained CE observed by nMOST, together with the non-uniform labelling of cholesterol by Filipin (Figure S3G), could suggest that lipid changes are driving structural rearrangements of the lipid-membrane in lysosomes. We therefore examined global phospholipids abundance, including PC and PE which are major building blocks for membranes. Indeed, *NPC2*^−/−^ cells accumulated LysoPCs under both fed and starvation conditions (Figure S4F), while LysoPE and other phospholipids did not show similar accumulation trajectories. Moreover, increased LysoPC appeared selective for NPC mutants, as LysoPC levels in *LIPA*^−/−^ or *GAA*^−/−^ cells were largely unchanged (Figure S4G). Lyso-PC species that were enriched in *NPC2*^−/−^ cells had, on average, <20-chain lengths than enriched PCs, potentially explaining the tight membrane stacks observed by cryo-ET (Figure S4H). As noted above, LysoPC is highly correlated with the accumulation of autophagy cargo receptors based on nMOST data (Figure S2E).

### Defective endocytic delivery to lysosomes in *NPC2*^−/−^ cells

We hypothesized that the multi-lamellar membrane nature of lysosomes in *NPC2*^−/−^ cells would result in an inability to fully deliver endocytic cargo to the lysosomal lumen, such that any cargo delivered by successful endosomal fusion with a defective lysosome would preferentially access space adjacent to the limiting membrane (Figured 4I). To examine this possibility, Control and *NPC2*^−/−^ cells under Fed conditions were incubated in the presence of extracellular endocytic cargo Dextran647 and lysotracker. Live cell imaging by structured illumination microscopy (SIM) revealed that while Dextran647 puncta in Control cells were largely co-incident with lysotracker, the Dextran647 signal in *NPC2*^−/−^ cells was largely excluded from lysotracker, and appeared to form partial “halo”-type structures surrounding lysotracker-positive puncta (Figure 4J). The simplest explanation for these results is that attempted fusion of Dextran647-laden endosomes with multilamellar lysosomes places Dextran647 signal co-incident with the limiting membrane without allowing full access to the lysosomal lumen (Figure 4I). As with LC3 and SQSTM1, much of the Dextran647 signal remained juxta-lysosomal (Figure 4J), consistent with defective fusion.

### Ferritin accumulation in NPC1 and NPC2 mutant cells

**Figure 5:**
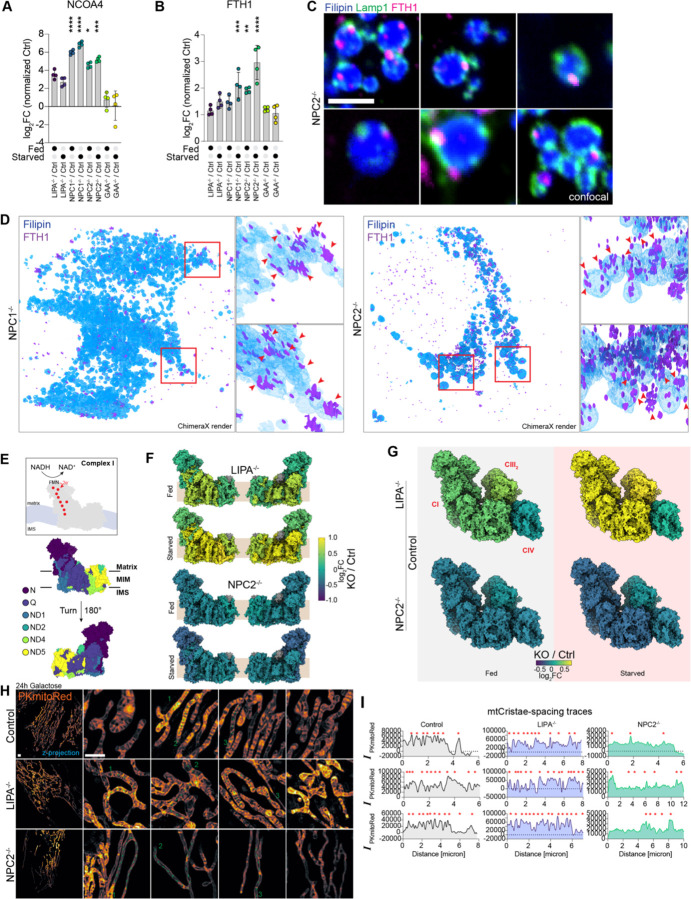
Ferritin accumulation and loss of mitochondrial cristae/OXPHOS systems in *NPC2*^−/−^ cells (see previous page). **(A,B**) Log_2_FC (mutant/Control) for FTH1 (A) and its autophagy adaptor NCOA4 (B) in Fed and starved cells as determined by nMOST. NCOA4: p(****) <0.0001, p(*) = 0.0244; FTH1: p(****) <0.0001; p(***) = 0.0007, p(**) = 0.0075. Data from quadruplicate replicates, ordinary one-way ANOVA with multiple comparisons, alpha = 0.05. Error bars depict S.D. **(C)** Confocal imaging of *NPC2*^−/−^ cells immunostained for α-LAMP1 and α-FTH1, with lysosomes marked by Filipin staining. A single z-slice is shown. Scale bar = 2 μm. **(D)** 3D-SIM reconstructions of *NPC1*^−/−^ or *NPC2*^−/−^ cells immunostained with α-FTH1 and the surface volume of cholesterol-rich lysosomes marked by Filipin. **(E)** Schematic of CI of the OXPHOS system with individual sub-modules indicated by the blue to yellow shading (PDB: 5XTH). **(F)** Log_2_FC of CI sub-module abundance in Fed and EBSS-treated conditions measured in *LIPA*^−/−^, *GAA*^−/−^, *NPC1*^−/−^, and *NPC2*^−/−^ cells by nMOST [normalized to control]. Based on quadruplicate replicate nMOST data. Legend shows color panel for log_2_FC values. **(G)** As in panel F but for the entire mitochondrial respirasome (CI, CIII_2_, CIV, PDB: 5XTH). Based on quadruplicate replicate nMOST data. **(H)** Z-projections of live-cell 3D-SIM images from Control, *LIPA*^−/−^ and *NPC2*^−/−^ cells after culturing on galactose (24 h) stained with the IMS dye PKmitoRed. Scale bar = 2 μm. **(I)** Line-plots of dashed lines from panel H of Control, *LIPA*^−/−^ and *NPC2*^−/−^ cells on 24h galactose growth conditions.

As noted above, we also observed a dramatic increase in the ferritinophagy regulator NCOA4^20,21^ in total proteome analysis (~log_2_ 3–7) when compared with Control or *GAA*^−/−^ cells (Figure 5A,B, Table S4). Increased NCOA4 was also observed previously in *NPC1*^−/−^ cells and associated with lysosomes.^17^ Ferritin, a cage-like protein complex composed of 24 copies FTH1 and/or FTL, binds ~4500 Fe^+3^ atoms and also promotes the conversion of Fe^+2^ species to Fe^+3^ to reduce reactivity.^29^ Ferritin cages are delivered to lysosomes in a process that requires NCOA4, which directly binds a conserved motif in FTH1 and promotes encapsulation within an autophagosome.^20,30–32^ Consistent with increased NCOA4 abundance, we also observed an increase in FTH1 abundance (log_2_FC ~1.5–1.8) under Fed conditions, and a further increase in abundance (log_2_FC ~2.0–3.0) under EBSS conditions in *NPC1*^−/−^ or *NPC2*^−/−^ cells (Figure 5A, Table S4). FTH1 abundance also correlated with LysoPC in lipid-proteome correlation analysis (Figure S2E).

Given the accumulation of LC3 and SQSTM1 juxtaposed with the lysosome in *NPC2*^−/−^ cells described above, we considered the possibility that FTH1 was also unable to access the lysosomal lumen. First, as expected based on proteomics, *NPC1*^−/−^ and *NPC2*^−/−^ cells displayed an increase in cytosolic FTH1 immunostaining when compared with Control or *GAA*^−/−^ cells and elevated FTH1 abundance was maintained in *NPC1*^−/−^ or *NPC2*^−/−^ cells treated with EBSS (Figure S5A,B). In single z-plane slices through *NPC2*^−/−^ cells, we observed FTH1-positive puncta preferentially localized to the periphery of Filipin-positive structures and FTH1 puncta were typically nearby but not co-incident with LAMP1-positive puncta (Figure 5C). Rendering of the full volume 3D-SIM images revealed numerous clusters of FTH1 puncta in the periphery of lysosomes, and in some cases a portion of FTH1-positive signal is co-incident with Filipin signal (Figure 5D). Taken together, these data suggest a block to turnover of multiple autophagy cargo receptors, as well as ferritin, at a step prior to or co-incident with autophagosomal fusion with lysosomes, rather than as a result of defects in degradation within the lysosomal lumen.^17^

### OXPHOS complex and cristae defects in *NPC2*^−/−^ cells

The finding that *NPC1*^−/−^ and *NPC2*^−/−^ cells exhibited accumulation of ferritin led us to explore possible iron dependent processes downstream of lysosomal function and ferritinophagy are affected. We analyzed nMOST data from *NPC1*^−/−^, *NPC2*^−/−^, *LIPA*^−/−^, *GAA*^−/−^, and Control cells in either Fed or EBSS-treated (6h) states for alterations in two systems that are heavily reliant on iron availability – cytosolic Fe-S cluster assembly machinery^33^ and components of the mitochondrial OXPHOS system, which contain several subunits with Fe-S clusters^34^. We were particularly interested in the OXPHOS system as GO terms related to this were found enriched in the LSD-nMOST screen for *NPC1*^−/−^ and NPC2^−/−^ cells (Figure S2E).^35^ Overall, the abundance of Fe-S cluster assembly machinery in *NPC1*^−/−^ and *NPC2*^−/−^ cells was largely unchanged when compared with Control cells (Figure S5C, Table S4). In contrast, when normalized to Control or *LIPA*^−/−^cells, we observed a reduction in a cohort of Complex I (CI) subunits in particularly in *NPC2*^−/−^ cells especially under starvation conditions, which was most pronounced for components of the N-module (log_2_FC ~−0.41) (Figure 5E–G, S5D–E). Three of five subunits within the N-module of CI contain Fe-S clusters, consistent with a reliance on iron for stability and/or assembly.^34,36^ The abundance of the N & Q-module, which contains two subunits with Fe-S clusters, as well as Complex IV (CIV), was also slightly reduced in *NPC2*^−/−^ mutants in the presence of EBSS (log_2_FC ~−0.1) (Figure 5E, S5D,E). The abundance of CI assembly factors in *NPC2*^−/−^ cells was similar to Control cells while CIV assembly factors in *NPC2*^−/−^ cells were increased ~2-fold (Figure S5F), suggesting that the reduced abundance of these complexes was not simply due to reduced levels of assembly factors themselves.

The intimate connectivity between OXPHOS complex assembly and mitochondrial cristae structure^37^, together with the finding that several MICOS-MIB complex components are reduced in *NPC2*^−/−^ cells relative to Control cells (in either Fed or EBSS conditions, Figure S5G), led us to examine mitochondrial ultrastructure. Utilizing a photo-stable and cell-permeable inner-membrane space (IMS) dye PKmitoRed, combined with live-cell 3D-SIM, we observed alterations in cristae morphology particularly in *NPC2*^−/−^ cells in the presence of galactose to enforce OXPHOS utilization (Figure 5H). Unlike Control cells, which displayed regularly spaced cristae, *NPC2*^−/−^ cells displayed an unexpected morphology reflective of altered cristae structure, including extensive regions of mitochondria, often near the cell periphery, that lacked obvious cristae as indicated by lineplots (Figure 5H,I; red stars indicate cristae bridge). For simplicity we refer to these regions as “cristae-deficient”. In contrast with *NPC2*^−/−^ cells, *LIPA*^−/−^ cells displayed an increased number of cristae, albeit with less regular intervals (Figure 5H,I), in line with the overall increase in OXPHOS and mitochondrial proteome compared to Control cells (Figure 5F, Figure S5H).

### Alleviation of cristae morphology defects in *NPC2*^−/−^ cells by extracellular iron

*NPC2*^−/−^ cells accumulate juxta-lysosomal FTH1, indicative of defects in delivery of iron-laden ferritin cages to the lysosome, where release of iron results in cytoplasmic transport to support the assembly of proteins harbouring Fe-S clusters. This phenotype correlated with alterations in mitochondrial cristae and formation of OXPHOS complexes in cells lacking NPC2. To examine whether mitochondrial defects could be mechanistically linked with iron availability, we tested whether extracellular iron delivered to the cytoplasm via Transferrin-dependent endocytosis, rather than ferritinophagy, could rescue cristae and OXPHOS defects. Transferrin-associated iron undergoes endocytosis, where the reduced pH of the endosome allows iron release from Transferrin and transport to the cytosol via the DMT1/SLC11A2 proton-coupled metal ion transporter.^38^

**Figure 6.**
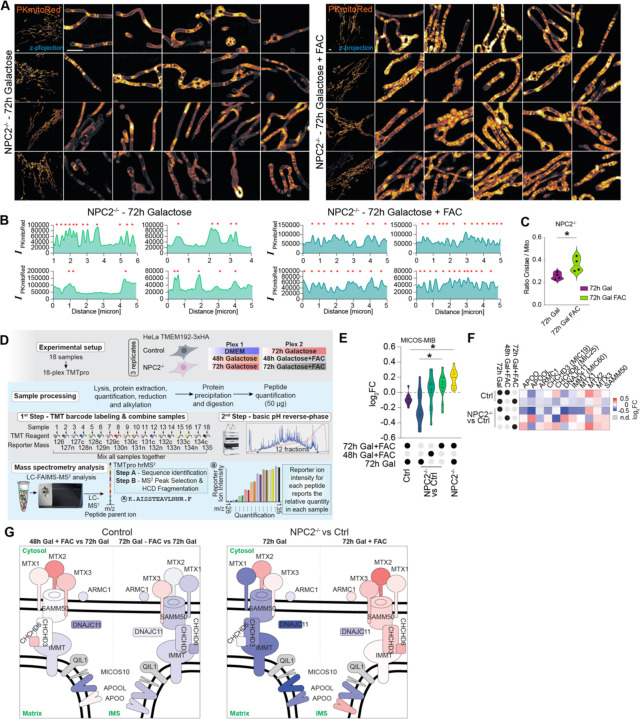
Rescue of mitochondrial cristae in *NPC2*^−/−^ cells by extracellular iron (see next page). **(A)** Z-projections of live-cell 3D-SIM images from Control and *NPC2*^−/−^ cells after culturing on Galactose (72 h) with or without FAC and stained with the IMS dye PKmitoRed. Scale bar = 2 μm. **(B)** Line-plots of individual mitochondria from panel A. Red asterisks indicate positions of cristae. **(C)** Violin plot depicting the ratio of cristae to mitochondria with and without FAC addition. Data based on 132 (72h Gal) or 148 (72h Gal + FAC) segmented planes of ROI-stacks from data shown in panel A; p(*) = 0.0242, unpaired t.test. **(D)** Schematic of TMT proteomics workflow for analysis of the effect of FAC addition to Control or *NPC2*^−/−^ cells. **(E)** Violin plots of log_2_FC [*NPC2*^−/−^/Control] of MICOS-MIB subunits in response to FAC. Data based on biological triplicate replicate TMTpro measurements; p(*) = 0.0162. **(F)** Heatmap of log_2_FC [*NPC2*^−/−^/Control] for individual MICOS-MIB subunits in response to FAC. Data based on triplicate biological replicate TMTpro measurements. **(G)** Schematic showing alterations in various MICOS-MIB subunits in response to FAC. Color coding is based on log_2_FC scale in panel F.

We first verified that FTH1 abundance, juxta-lysosomal FTH1 localization, and endolysosomal system fusion phenotypes are retained in *NPC2*^−/−^ cells grown in Galactose (Figure S6A,B). We then examined cristae morphology in *NPC2*^−/−^ cells grown in Galactose in the presence or absence of FAC (ferric ammonium citrate, 72h) as an extracellular iron source. Remarkably, both the frequency of cristae and their spacing as quantified by lineplots were substantially rescued by FAC addition (Figure 6A–C, Figure S5I). We also examined (ΔΨm) in *NPC2*^−/−^ and Control cells by determining the ratio of Mitotracker-DeepRed (mtDR) to Tetramethylrhodamine Methyl Ester (TMRM). As expected, Control cells grown on galactose (72h) displayed high membrane potential, as indicated by the prominent TMRM signal (Figure S6C,D). In contrast, *NPC2*^−/−^ cells display reduced membrane potential, as represented by an increase in the ratio of intensities (*I*[mtDR/TMRM]) with many cells exhibiting low ΔΨm (Figure S6C). Consistent with rescue of cristae with FAC added to the media, ΔΨm was enhanced upon growth in FAC and cell growth was increase by ~ 28% (Figure S6C,E). Taken together, these data indicate that these aspects of mitochondrial dysfunction in *NPC2*^−/−^ cells are substantially alleviated by providing a ferritinophagy-independent route for iron delivery to the cytosol and mitochondria.

### MICOS-MIB complex proteome remodelling in *NPC2*^−/−^ cells by extracellular iron

To examine the effect of FAC addition on the mitochondrial proteome in an unbiased manner, we performed two 18-plex TMT proteomics experiments examining total proteomes from Control or *NPC2*^−/−^ cells under 5 conditions: DMEM, 48h Galactose +/− FAC, 72h Galactose +/− FAC (Figure 6D, Table S5). Importantly, GO analysis of *NPC2*^−/−^-dependent alterations in the cellular proteome (72h in Galactose) yielded terms linked with lysosomal function, intracellular iron accumulation, and iron binding (Figure S6F). Moreover, we observed the expected increase in the abundance of LC3B, SQSTM1, and TAX1BP1 as well as NCOA4, FTH1 and FTL (Figure S6G–I). Overall, these data validate the results of the nMOST proteomic analysis and also indicate that the major phenotypes seen upon NPC2 deletion under Fed conditions described above are maintained when cells are grown on Galactose.

We next examined the mitochondrial proteome. The overall abundance of the mitochondrial proteome was largely unchanged in Control cells treated with FAC for 48 or 72h and in *NPC2*^−/−^ cells with 48h of FAC treatment but was slightly increased in *NPC2*^−/−^ cells at 72h; however, classifying protein abundance based on their mitochondrial sub-compartment revealed differential changes in response to Galactose and FAC addback (Figure S6J,K). Indeed, *NPC2*^−/−^ cells grown in Galactose (72h) displayed a reduction in the abundance of MICOS-MIB complex components when compared with Control cells (Figure 6E,F), in accordance with the observed reduction in cristae (Figure 5H,I; Figure 6A). In contrast, the abundance of MICOS-MIB complex subunits was largely rescued by FAC for either 48 or 72h, in line with the microscopy data (Figure 6E,F). Alterations in the abundance of individual MICOS subunits are displayed schematically in Figure 6G. Taken together, these results indicate that imbalances in iron-homeostasis can lead to reversible changes of the mitochondrial ultrastructure.

### OXPHOS complex proteome remodelling in *NPC2*^−/−^ cells by extracellular iron

**Figure 7.**
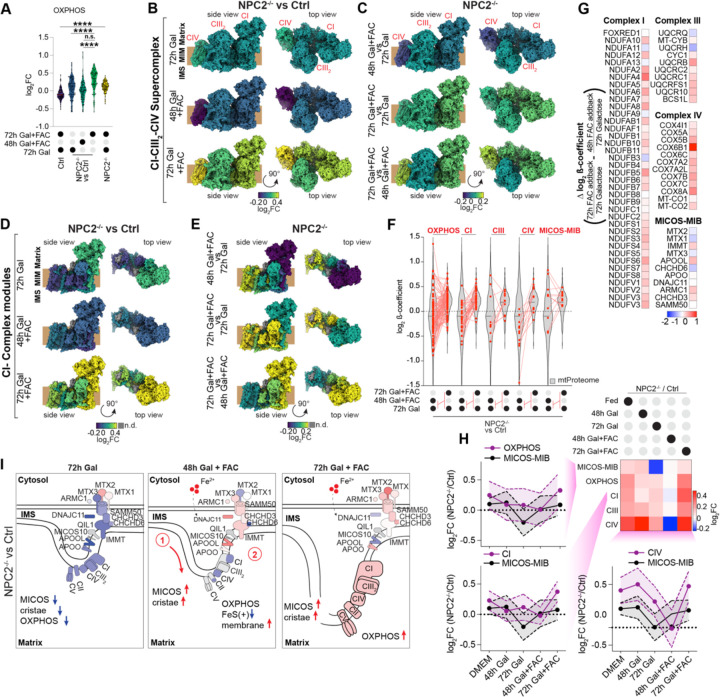
Rescue of OXPHOS complex abundance in *NPC2*^−/−^ cells by extracellular iron (see next page). **(A)** Violin plot of OXPHOS subunit log_2_FC values in *NPC2*^−/−^ versus Control cells grown in Galactose with or without FAC. p(****) < 0.0001, ordinary one-way ANOVA with multiple comparisons, alpha = 0.05; data based on triplicate biological replicate TMTpro measurements. **(C,B)** Log_2_FC of supercomplex abundance in Galactose with or without FAC addback for *NPC2*^−/−^ versus Control cells. Legend shows color panel for log_2_FC values. Data based on triplicate biological replicate TMTpro measurements. **(D,E)** Log_2_ FC of CI abundance in Galactose with or without FAC addback for *NPC2*^−/−^ versus Control cells. Legend shows color panel for log_2_FC values. Data based on triplicate biological replicate TMTpro measurements. (**F**) Log_2_ β -coefficients for the indicated treatments shown for all OXPHOS subunits and individual sub-complexes (from Figure 6D). **(G)** Heatmaps for log_2_ β-coefficient [(72h FAC addback/72h Galactose) – (48h FAC addback/72h Galactose)] from indicated OXPHOS subunits in the total proteome from Figure 6D. Data based on triplicate biological replicate TMTpro measurements. **(H)** Log_2_FC [*NPC2*^−/−^/Control] for the indicate protein complexes under the indicated conditions (time in Galactose with or without FAC addback). Data based on triplicate biological replicate TMTpro measurements. **(I)** Schematic model for the rescue of mitochondrial cristae and OXPHOS complexes upon FAC addback in *NPC2*^−/−^ cells. See text for details.

We next turned to components of the OXPHOS system, embedded in the mitochondrial cristae and whose relative abundance was reduced in *NPC2*^−/−^ cells (Figure S5D,E). First, when viewed across all OXPHOS subunits as a cohort, an overall increase in abundance can be seen over the FAC addback time-course (Figure 7A, Table S5). We next parsed the dataset based on subcomplexes of the respirasome (CI-CIII_2_-CIV) and their respective assembly factors (Figure S7A–C). A linear increase in CI and CIII abundance was observed upon FAC addback when comparing [*NPC2*^−/−^/Ctrl], which was co-incident with increased levels of respective assembly factors. However, CIV displayed a bi-phasic pattern with an initial decrease in abundance at the 48h time-point.

We mapped the alterations in abundance in response to FAC onto the OXPHOS supercomplex (CI-CIII_2_-CIV) and CI structures in the context of *NPC2*^−/−^ versus Control cells (Figure 7B,D) or *NPC2*^−/−^ cells alone (Figure 7C,E). Focusing on CI, changes in N- and Q-modules, major sites of FeS cluster-containing protein occurrence, revealed the largest shifts in presence of FAC. Interestingly, at early time-points these modules appear de-stabilized, followed by stabilization of the membrane-arm modules (ND1,2,4,5) and subsequent increase in N-module proteins between 48 and 72h FAC addback (Figure 7D,E). Next, we evaluated the effect the growth media conditions and FAC addback has on the proteome in the context of *NPC2*^−/−^ vs Control (β-coefficient, Figure S7D). Log_2_ β-coefficients for the transition from 48 to 72h in the presence of FAC and Galactose did not affect the proteome at large (β-coefficient = 0.008); however, although individual subunits displayed differential alterations in abundance, generally detectable increases in mitochondria (β-coefficient = 0.16), and especially OXPHOS components (β-coefficient = 0.21) were observed. This included all but one subunit of N- and Q-modules, as well as all the nuclear genome-encoded CIV subunits (Figure 7F,G). Consistent with the differences observed on the abundance between 48 and 72h, β-coefficients across OXPHOS components at these time-points revealed differential changes that were not see with MICOS-MIB subunits (Figure S7E). Interestingly, recovery of CI and CIV subunits primarily occurred during the 48 to 72h interval and was proceeded in the timing by MICOS-MIB rescue (Figure 7H). We also examined alterations the abundance of proteins known to function in assembly of Fe-S clusters in either the cytoplasm or the mitochondria (Figure S6H). The log_2_FC *NPC2*^−/−^/Control values of most cytosolic Fe-S cluster assembly (CIA) components^39^ were slightly decreased in response to 72h of FAC, while, in contrast, the abundance of mitochondrial Fe-S cluster (ISC) assembly proteins were either increased or remained constant, with the exception of ABCB7, which functions to transport [2Fe-2S]-(Glutathione)4 from the mitochondria to the cytosol.^39^ These data are consistent with an elevated Fe-S cluster biogenesis pathway in mitochondria of *NPC2*^−/−^ cells treated with FAC when compared with Control cells.

## DISCUSSION

Here we have developed the nMOST workflow for simultaneous analysis of lipids and proteins from the same sample and have applied this workflow to a collection of more than two dozen cell lines lacking individual LSD genes. Cross-correlation analysis between lipids and proteins across various genotypes reveals numerous molecular phenotypes associated with specific LSD alleles, and the data provided here provides a resource for further mechanistic discovery.

We observed a prominent and selective phenotype with NPC1, NPC2 and TPP1 mutants involving accumulation of autophagy regulators, which correlated with accumulation of LysoPC. Through 3D-SIM imaging and cryo-ET, we provided evidence for a block in autophagic clearance wherein autophagic receptors (e.g. LC3B) accumulate in juxta-lysosomal locations with evidence of defective delivery of cargo to the lysosomal lumen. This phenotype, which was more pronounced in *NPC2*^−/−^ cells than *NPC1*^−/−^ cells, correlated with the formation of multilamellar lysosomes, which may be reflective of the increased abundance of LysoPC in these cells (Figure 2C). Previous studies^18,40^ have implicated decreased lysosomal cleavage of cargo as well as defects in autophagosome-lysosome fusion, but the underlying mechanisms were unclear. We suggest that multilamellar membranes within lysosomes observed by cryo-ET reduce the ability of lysosomes to efficiently fuse with either autophagosomes or endosomes, thereby limiting delivery of cargo to the lysosomal lumen.

Among the autophagic cargo that accumulated in *NPC1*^−/−^ and *NPC2*^−/−^ cells was the ferritin cage protein FTH1, which was juxta-lysosomal based on 3D-SIM imaging. Given that a block to ferritin degradation in the lysosome would be expected to reduce iron availability, we examined complexes known to rely on Fe-S clusters for their production, leading to the identification of mitochondrial electron transport chain complexes as being reduced in cells lacking NPC2 (Figure 7I, left panel). Loss of OXPHOS complexes correlated with reduced cristae number and MICOS-MIB complexes in *NPC2*^−/−^ cells ((Figure 7I, left panel). Remarkably, delivery of iron to cells through endocytosis results in initial accumulation of MICOS-MIB subunits at 48h (Figure 7I, middle panel), which supports further assembly of OXPHOS complexes at 72h, with near full restoration of OXPHOS complexes and cristae number (Figure 7I, right panel). The behaviour of MICOS-MIB and OXPHOS complexes and the effects on cristae number are consistent with the self-reinforcing role that these components play in formation and stabilization of cristae.^37^ We note that while cells lacking *NPC1* also accumulate FTH1, the corresponding phenotypes and in particular the mitochondrial alterations seen in *NPC2*^−/−^ cells appear more pronounced, despite only 5% of NPC patients carry mutations in the *NPC2* gene.^41^ Previous studies have described an imbalance of iron metabolism and haematological abnormalities in *NPC1* mouse models and in patients with Niemann-Pick disease type C1.^42^ Further studies are required to understand the extent to which an inability to promote iron mobilization by autophagy and concomitant effects on mitochondrial function are linked with defects observed in patients. The platform we have described here and its application to relevant cell lineages linked with LSDs may facilitate identification of molecular defects or pathways with relevance to disease.

## LIMITATIONS OF THE STUDY

First, our collection of HeLa cells lacking LSD genes is incomplete, and further studies are required to obtain and characterize the full set LSD mutants. In addition, while cancer cell lines such as HeLa are known to display phenotypes in common with more physiologically relevant cell system, including for example loss of *GRN* and its effect on lipids,^4^ analysis of a broader array of cell types is required to understand the generality of lipidomic and proteomic phenotypes reporter here.

## STAR METHODS

All details and catalogue numbers can be found in the Reagents and Tools Table.

### Cell culture

HeLa^TMEM192-HA^ cells^22^ were maintained in Dulbecco’s modified Eagle’s medium (DMEM), supplemented with 10 % vol/vol fetal bovine serum (FBS), 5 % vol/vol penicillin-streptomycin (P/S), 5 % vol/vol GlutaMAX and 5 % vol/vol non-essential amino acids (NEAA) at 37°C, 5 % O_2_. Unless otherwise noted, we refer to independently grown and handled cultures as biological replicates to distinguish from assays performed on identical samples (i.e. technical replicates). For galactose growth conditions, galactose-containing DMEM was prepared from Glucose-free DMEM supplemented with 10 % vol/vol dialysed FBS, 25 mM D-galactose, 5 % vol/vol penicillin-streptomycin (P/S), 5 % vol/vol GlutaMAX), 5 % vol/vol Sodium Pyruvate and 50 μg/mL Uridine.

### Gene-Editing

Generation of LSD mutants in the HeLa^TMEM192-HA^ background^22^ was facilitated using CRISPR/Cas9 with target sites determined using CHOPCHOP.^43^ Guide RNAs were ligated into the px459 plasmid (Addgene plasmid # 62988) and cells transfected using Lipofectaime LTX reagent (Thermo Fisher Scientific, 15338100), according to manufacturer’s instructions. Two days post-transfection, single, puromycin-resistant cells were sorted into 96-well dishes containing 300 μL full growth medium (composition as described above). Single cells were allowed to grow into colonies and duplicated for multiplex sequencing. Genomic DNA samples were obtained by incubating cells in 30 μL PBND (50 mM KCl, 10 mM Tris-HCl, pH 8.3, 2.5 mM MgCl2–6H_2_O, 0.45% NP-40 and 0.45% Tween-20) with protease K (40 μg/ml) at 37°C for 5 min and heated to 55°C and 95°C for 30min and 15 min, respectively. The first round of PCR was performed to amplify the target region using gene-specific primers that contain partial Illumina adaptor sequences (i.e., Forward primer: 5’-ACACTCTTTCCCTACACGACGCTCTTCCGATCT[n]18–22 -3’, Reverse primer: 5’-GTGACTGGAGTTCAGACGTGTGCTCTTCCGATCT[n]18–22 -3’, [n]18–22 represent gene specific sequences). The resulting PCR products with adapter-modified ends can be further amplified in the second round of PCR by universal primers containing attachment sites for the flow cell and index sequences (i.e., Forward primer: 5’-AATGATACGGCGACCACCGAGATCTACACTCTTTCCCTACACGACGCTCTTCCGATCT -3’, Reverse primer: 5’-CAAGCAGAAGACGGCATACGAGAT-[n]8-GTGACTGGAGTTCAG ACGTGTGCT -3’, [n]8 represents index sequences). The final PCR products were purified using QIAquick PCR purification kit (Qiagen, 28106). Sequencing was performed using Miseq Reagent kits v2 on Illumina Miseq following the denature and dilute libraries guide of Miseq system, and sequencing data was analysed by Outknocker program (www.OutKnocker.org).^44^ Knockout candidates were confirmed by Western blot on whole cell lysates or by proteomics. The sgRNAs were generated using GeneArt Precision gRNA Synthesis Kit (Thermo Fisher Scientific) according to the manufacturer’s instruction and purified using RNeasy Mini Kit (Qiagen). The sgRNA target sequences and sequencing results can be found in Table S1. The HeLa TMEM192–3xHA Control (referred to as HeLa^TMEM192-HA^), *GRN*^−/−^ and *HEXA*^−/−^ have been previously reported.^4,22^

## PROTEOMICS

### Sample Preparation for nMOST

For samples used for technical evaluation of MOST, Bead-enabled, Accelerated, Monophasic Multi-omics (BAMM) method was used.^14^ Silica coated superparamagnetic beads (700 nm, SeraSil-Mag) were washed and resuspended in water for a concentration of 75 μg/μL, while frozen cell pellets were being thawed on ice. 200 μL acetonitrile (ACN), 600 μL n-butanol, and 200 μL beads containing water were added to samples. After vortex, samples were sonicated for 5 min at 14°C. Beads were immobilized by magnet and 100 μL supernatant was aliquoted, dried down, and reconstituted in 300 μL n-butanol:isoproponal (IPA):water (8:23:69, v/v/v) in an amber autosampler vial for lipids.^45,46^ The remaining supernatant was removed. The beads were reconstituted in Rapid Digestion Buffer (Promega) diluted to 75% by water with 2 mM TCEP and 40 mM CAA. After incubation for 10 min at room temperature, trypsin (Promega) was added in a 20:1 ratio (protein-to-trypsin). The samples were incubated in thermomixer for 40 min at 60 °C and 1000 RPM. Formic acid was added to terminate digestion. Peptides were desalted by Sep-Pak (Waters) C18 column, dried down in SpeedVac (Thermo Fisher Scientific), and reconstituted in 0.2% formic acid.

For HeLa whole cell extracts or Lyso-IP samples (generated as described^4,22^; dx.doi.org/10.17504/protocols.io.ewov14pjyvr2/v2.) samples, 300 μL mixture of n-butanol:ACN:water (3:1:1, v/v/v) was added. Samples were bath sonicated for 5 min at 14°C. After centrifugation at 14000 g for 5 min, 50 μl of the lipid containing supernatant was transferred to autosampler vials with glass insert, dried down in speedVac, and resuspend in 50 μL n-butanol:IPA:water (8:23:69, v/v/v).^45,46^ The remaining samples were maintained at −80°C until protein digestion. For protein digestion of whole cell extracts, the samples were thawed on ice and centrifuged at 14000 g for 5 min. The remaining supernatant was removed from samples. 100 μL lysis buffer (8 M Urea, 100 mM Tris pH 8.0, 10 mM TCEP, 40 mM CAA) was added. The samples were bath sonicated for 5 min at 14°C and vortexed for 15min. Protein concentration was determined by Thermo protein BCA assay (reducing agent compatible). LysC (FUJIFILM Wako) was added to samples in a 50:1 ratio (protein-to-LysC) and incubate on a rocker for 4 h at room temperature. The urea was diluted to 2 M by 300 μL 100 mM Tris pH 8.0. Trypsin was added to samples in a 50:1 ratio (protein-to-trypsin) and incubate on a rocker overnight at room temperature. For protein digestion of Lyso-IP samples, 60 μL 6M GnHCl, 100mM Tris was added to the sample to solubilize proteins from being aggregated on beads. The samples were bath sonicated 5 min at 14 °C; incubated in thermomixer for 5 min at 100 °C and 600 RPM, and then incubated for 2 h at 80 °C and 600 RPM. Beads were immobilized by magnet and supernatant was transferred to a 96well plate. GnHCl was diluted to 2M by adding 120μL 100mM Tris, 10 mM TCEP, 40 mM CAA. LysC was added to samples in a 50:1 ratio (protein-to-LysC) and incubate on a rocker for 4h at room temperature. GnHCl was diluted to 0.4M by adding 420 μL 100mM Tris pH8.0. Trypsin was added to samples in a 50:1 ratio (protein-to-trypsin) and incubate on a rocker overnight at room temperature. 10 % TFA was added to terminate digestion. After centrifugation at 12000 g for 5 min, digested peptides were desalted by StrataX 10 mg 96-well Plate (Phenomenex), dried down in SpeedVac, and reconstituted in 0.2 % formic acid. The peptide concentration was determined by Thermo peptide BCA assay.

### nMOST LC-MS

Separation was performed on an in-house packed BEH C18 capillary column (28 cm length × 75 μm inner diameter × 1.7 μm particle size) at 60 °C and an Ultimate3000 system (Thermo Scientific). Column packing was described previously.^47^ Mobile phase A consisted of 0.2% formic acid in water. Mobile phase B consisted of 0.2% formic acid and 5 mM ammonium formate in IPA/ACN (90:10, v/v). Lipids were loaded onto column first and then peptides at 0% mobile phase B. Mobile phase B increased to 70% over 80 min for scanning MS/MS spectra of peptides, increased to 100% over 26 min for scanning MS/MS spectra of lipids. The column was washed at 100% mobile phase B for 3 min and re-equilibrated at 0% mobile phase B for 10 min. Eluting analytes were analyzed by an Orbitrap Eclipse Tribrid mass spectrometer (Thermo Scientific). Spray voltage was 2 kV. Ion transfer tube temperature was 275°C. MS^1^ scan range was 200–1,600 *m/z*. MS^1^ resolution was 240,000 (at 200 *m/z*). Source RF was 35. MS^1^ AGC target was 300%. MS^1^ injection time was 50 ms. Duty cycle was 1 s. Polarity was positive. For proteomics data acquisition from 0 to 80 min, precursor selection range was 300–1,350 *m/z*. Charge states were 2–5. Dynamic exclusion was 10 s. Isolation width was 0.5 *m/z*. Precursors were fragmented by higher-energy collisional dissociation (HCD) with a normalized collision energy (NCE) of 25%. MS^2^ mass spectra were acquired in data-dependent mode using ion trap turbo speed. MS^2^ scan range was 150–1,350 *m/z*. MS^2^ AGC target was 300%. MS^2^ injection time was 14 ms. For lipidomics data acquisition from 80 to 120 min, precursor selection range was 300–1,600 *m/z*. Charge states were 1–2. Dynamic exclusion was 10 s. Isolation width was 0.7 *m/z*. Precursors were fragmented by higher-energy collisional dissociation (HCD) with a stepped NCE of 27% ± 5%. MS^2^ mass spectra were acquired in data-dependent mode using ion trap rapid speed. MS^2^ scan range was auto. MS^2^ AGC target was 300%. MS^2^ injection time was 17 ms. Real-time library search (RTLS) and complementary collision-induced dissociation (CID) were used for glycerophospholipids and sphingomyelins as described previously.^15^ For large scale LSD samples, to improve the throughput of the analysis, the total LC time was set down to 105 min. Peptides eluted and were analyzed from 0 to 70 min while lipids eluted and were analyzed from 70 to 105 min.

### nMOST MS Data Process

For proteomics, raw data files were processed by MaxQuant (Version 2.0.3.0). The database was canonical plus isoforms downloaded from Uniprot in December 2021. The match between runs was on. MS/MS spectra were not required for LFQ comparisons. For lipidomics, raw data files were processed using Compound Discoverer 3.1 (Thermo Scientific) and Lipidex.^48^ Peak detection required a signal-to-noise ratio of 1.5, a minimum peak intensity of 5 × 10^5^, and a maximum peak width of 0.75 min. The chromatographic peaks were grouped into compound groups by a retention time tolerance of 0.5 min and a mass tolerance of 10 ppm. Peaks were removed if the peak areas of sample over blank were < 3-fold. An in silico generated lipid spectral library (LipiDex_HCD_Formic) was used for MS/MS spectra searching. The threshold of dot product score was 500 and the threshold of reverse dot product score was 700. MS^2^ spectra were annotated at the molecular species level if the minimum spectral purity was at least 75%; otherwise, sum compositions were reported. The lipid identification was further filtered for adducts, dimers, in-source fragments, misidentified isotopes, and mismatched retention time by LipiDex and the degreaser module of LipiDex 2 (https://github.com/coongroup/LipiDex-2). Cross-ome correlation analysis between lipids and proteins analysed by nMOST. Proteins and lipids were correlated using Kendall rank correlation approach (R function corr(); the resulting matrix was filtered for lipids or proteins with at least 2 correlations |>0.4| Tau. The filtered matrix was further clustered using hierarchical clustering and subsetted in to 18 protein clusters and 14 lipid cluster (kmeans). Members of each cluster were evaluated for enrichment in GO terms (Cellular components) or lipids class using a fisher’s exact test.

### TMTpro 18plex proteomics

#### Proteomic sample preparation.

Sample preparation of proteomic analysis of whole-cell extract from HeLa control and mutant lysates performed according to previously published studies.^49,50^ Replicate cell cultures were grown and treated independently and are considered biological replicates in the context of TMT experiments. Cells were washed twice with 1xPBS and harvested on ice using a cell scraper in 1xPBS. Cells were pelleted via centrifugation for 5 min (5000*g*, 4°C), and washed with 1xPBS before resuspending in lysis buffer (Urea, 150 mM TRIS pH 7.4, 150mM NaCl, protease and phosphatase inhibitors added). After 10 second sonication, and optional French-pressing through a G25 needle, lysed cells were pelleted and protein concentration of clarified sample determined using BCA kit (Thermo Fisher Scientific, 23227). 100 μg protein extract of each samples were incubated for 30 min @ 37°C with 5 mM TCEP for disulfide bond reduction with subsequent alkylation with 25 mM chloroacetamide for 10 min at RT with gentle shaking. Methanol-chloroform precipitation of samples was performed as follows: To each sample, 4 parts MeOH was added, vortexed, one part chloroform added, vortexed, and finally 3 parts water added. After vortexing, suspension was centrifugated for 2 min at 14000*g* and the aqueous phase around the protein preticipate removed using a loading tip. Peptides were washed twice with MeOH and resuspended in 200 mM EPPS, pH 8, and digested for 2h with LysC (1:100) at 37°C, followed by Trypsin digestion (1:100) at 37°C overnight with gentle shaking.

#### Tandem mass tag (TMT) labeling.

50 μL of digested samples were labeled by adding 10 μL of TMT reagent (stock: 20 mg/ml in acetonitrile, ACN) together with 10 μL acetonitrile (final acetonitrile concentration of approximately 30 % (v/v)) for 2h at room temperature before quenching the reaction with hydroxylamine to a final concentration of 0.5 % (v/v) for 15 min. The TMTpro-labeled samples were pooled together at a 1:1 ratio, resulting in consistent peptide amount across all channels. Pooled samples were vacuum centrifuged for 1h at room temperature to remove ACN, followed by reconstitution in 1 % FA, samples were desalted using C18 solid-phase extraction (SPE) (200 mg, Sep-Pak, Waters) and vacuum centrifuged until near dryness.

#### Basic pH reverse phase HPLC.

Dried peptides were resuspended in 10 mM NH_4_HCO_3_ pH 8.0 and fractionated using basic pH reverse phase HPLC.^51^ Samples were offline fractionated into 96 fractions over a 90 min run by using an Agilent LC1260 with an Agilent 300 Extend C18 column (3.5 μm particles, 2.1 mm ID, and 250 mm in length) with mobile phase A containing 5 % acetonitrile and 10 mM NH_4_HCO_3_in LC-MS grade H_2_O, and mobile phase B containing 90 % acetonitrile and 10 mM NH_4_HCO_3_ in LC-MS grade H_2_O (both pH 8.0). The 96 resulting fractions were then pooled in a non-continuous manner into 24 fractions.^52^ This set of 24 fraction was divided into 2x12 sets (even or odd numbers), acidified by addition of 1 % Formic Acid (FA) and vacuum centrifuged until near dryness. One set (12 samples) was desalted via StageTip, dried and reconstituted in 10 μL 5 % ACN, 5 % FA before LC-MS/MS processing.

#### Mass spectrometry acquisition.

For HeLa whole-cell proteomics, data collection was performed on a Orbitrap Fusion Lumos Tribrid mass spectrometer (Thermo Fisher Scientific, San Jose, CA), coupled with a FAIMS Pro device and a Proxeon EASY-nLC1200 liquid chromatography (Thermo Scientific). 10 % of resuspended samples were loaded on a 35 cm analytical column (100 mm inner diameter) packed in-house with Accurcore150 resin (150 Å, 2.6 mm, Thermo Fisher Scientific, San Jose, CA) for LC-MS analysis. Peptide separation was performed with a gradient of acetonitrile (ACN, 0.1 % FA) from 3–13 % (0–83 min) and 13–28 % (80–83 min) during a 90 min run. LC-MS/MS was combined with 3 optimized compensation voltages (CV) parameters on the FAIMS Pro Interface to reduce precursor ion interference.^53^ Data-dependent acquisition (DDA) was performed by selecting the most abundant precursors from each CV’s (−40/−60/−80) MS1 scans for MS/MS over a 1.25 s duty cycle. The parameters for MS1 scans in the Orbitrap include a 400–1,600 m/z mass range at 60,000 resolution (at 200 Th) with 4 x 105 automated gain control (AGC) (100 %), and a maximum injection time (max IT) of 50 ms. Most abundant precursors (with 120 s dynamic exclusion +/− 10 ppm) were selected from MS1 scans, isolated using the quadrupole (0.6 Th isolation), fragmented with higher-energy collisional dissociation (HCD, 36 collision energy), and subjected to MS/MS (MS2) in the Orbitrap detector at 50,000 resolution, 5x AGC, 110 – 200 m/z mass range, IT 86 ms and with 120 s dynamic exclusion +/− 10 ppm.

#### Data processing.

Raw mass spectra were converted to mzXML, monoisotopic peaks reassigned using Monocle^54^ and searched using Comet^55^ against all canonical isoforms found in the Human reference proteome database (UniProt Swiss-Prot 2019–01; https://ftp.uniprot.org/pub/databases/uniprot/previous_major_releases/release-2019_01/)) as well as against sequences from commonly found contaminant proteins and reverese sequences of proteins as decoys, for target-decoy competition.^56^ For searches, a 50-ppm precursor ion tolerance and 0.9 Da product ion tolerance for ion trap MS/MS as well as trypsin endopeptidase specificity on C-terminal with 2 max. missed cleavages was set. Static modifications were set for carbamidomethylation of cysteine resiudes (+57.021 Da) and TMTpro labels on lysine residues and N-termini of peptides (+304.207 Da); variable modification was set for oxidization of methionine residues (+15.995 Da). Peptide-spectrum matches were filtered at 2 % false discovery rate (FDR) using linear discriminant analysis (Picked FDR method, based on XCorr, DeltaCn, missed cleavages, peptide length, precursor mass accuracy, fraction of matched product ions, charge state, and number of modifications per peptide (additionally restricting PSM Xcorr >1 and peptide length>6,^57^ and after a 2 % protein FDR target filtering^58^ PSM reporter ion intensities were quantified. Quantification was performed using a 0.0003-Da window around the theoretical TMT-reporter m/z, and filtered on precursor isolation specificity of > 0.5 in the MS1 isolation window and for CORE output filtered by summed SNR across all TMT channels > 100. MS statsTMT^59^ was performed on peptides with >200 summed SNR across TMT channels. For each protein, the filtered peptide–spectrum match TMTpro raw intensities were summed and log_2_ normalized to create protein quantification values (weighted average) and normalized to total TMT channel intensity across all quantified PSMs (adjusted to median total TMT intensity for the TMT channels).^60^ Log_2_ normalized summed protein reporter intensities were compared using a Student’s t-test and p-values were corrected for multiple hypotheses using the Benjamini-Hochberg adjustment.^61^ Linear model analysis was performed as described.^62^ Subcellular and functional annotations were based on previous published list of high confidence annotations (^63^, “high” & “very high” confidence, additional manual entries from^49^, AmiGO Pathway online tool and mitochondrial annotation was based on MitoCharta 3.0^64^). Part of heatmaps were created using Morpheus (https://software.broadinstitute.org/morpheus).

## MICROSCOPY

Macros and pipelines used in this work can be found on GitHub (https://github.com/harperlaboratory/LSDnMOST)

### Live-cell spinning disk microscopy – general acquisition parameters

For analysis of organelles using live-cell spinning disk microscopy, cells were seeded into either 24-well 1.5 high performance glass bottom plates (Cellvis, P24–1.5H-N) or μ-Slide 8-well, glass bottom plates (ibidi, #80807) and further cultured in the vessel until reaching appropriate confluency for microscopy. Before microscopy, cells were washed in 1x PBS and imaged in FluoroBrite DMEM media. Cells were imaged using a Yokogawa CSU-X1 spinning disk confocal on a Nikon Eclipse Ti-E motorized microscope. The system is equipped with a Tokai Hit stage top incubator and imaging was performed at 37°C, 5 % CO_2_ and 95 % humidity under a Nikon Plan Apo 60×/1.40 N.A immersion oil objective lens. Fluorophores were excited in sequential manner with a Nikon LUN-F XL solid state laser combiner ([laser line – laser power]: 405 (80mW), 488 (80 mW), 561 (65 mW), 640nm (60 mW)]) using a Semrock Di01-T405/488/568/647 dichroic mirror. Fluorescence emissions were collected through a Chroma ET455/50m [405 nm], Chroma ET525/36m [488 nm], Chroma ET 605/52m [561nm] and a Chroma ET700/75m [for 640 nm] filters, respectively (Chroma Technologies). Images were acquired with a Hamamatsu ORCA-Fusion BT CMOS camera (6.5 μm^2^ photodiode, 16-bit) camera and NIS-Elements image acquisition software. Consistent laser intensity and exposure time were applied to all the samples, and brightness and contrast were adjusted equally by applying the same minimum and maximum display values in ImageJ/FiJi^65^. Image quantification was performed in ImageJ/FiJi using custom-written batch-macros.

### Live-cell microscopy for mitochondrial membrane potential measurements

For measuring mitochondrial membrane potential in live-cells, HeLa^TMEM192-HA^ control and mutant cell lines were seeded in μ-Slide 8 well chambers and treated according to the experimental plan. Before imaging, cells were incubated with TMRM (1:5000) and MitoTrackerDeepRed (1:10000) for one hour at 37°C, washed twice with PBS and growth media replaced before imaging. 5 % laser power and 100 ms (568 nm) or 50 ms (640 nm) exposure time was used to image 6 μm z-stacks of cells. Mitochondrial masks were created based on the MitoTracker-DeepRed signal and TMRM intensities measured within these masks for evaluation.

### Measurement of lysosomal pH using live-cell spinning disk microscopy

Day before measurements, 100,000 cells were seeded in 24-well glass bottom plate (Cellvis). On the day of measurement, cells were loaded with SiR-Lysosome (1:1000, Cytoskeleton Inc.) and pHLys Red (1:1000, Dojindo) for 1h in DMEM + 10 % FBS. Stains was then washed out and chased with phenol-red free DMEM+10 % FBS for 3h before imaging. For BafA1 treatment, 1 μM BafA1 was treated 2.5 hours into the chase for 30 minutes prior to live-cell imaging on confocal microscope with 20x objective. To establish the pH calibration curve, wildtype cells were bathed in calibration buffers with pH adjusted to 3, 4, 5, 6, and 7, supplemented with 10 μM monensin.^66^ For both experimental and pH calibration conditions, 5 to 6 field of views were imaged and analyzed in its entirety. This process was repeated for each independent experiment. Image analysis was performed using Fiji (ImageJ). For each field of view, background subtraction was processed using the rolling ball background subtraction method for each channel. Subsequently, Otsu’s method was used to threshold the SiR-Lysosome signal to select region of interest (ROI) corresponding to lysosomes. The selected ROI was applied to the pHLys Red channel and then measured fluorescence intensity. The fluorescence intensity of the pHLys Red channel was then fitted to the calibration curve to calculate pH value.

### Immunocytochemical analysis

HeLa cells were fixed with warm 4 % paraformaldehyde (Electron Microscopy Science, #15710, purified, EM grade) in PBS at 37°C for 30 min and permeabilized with 0.5 % Triton X-100 in PBS for 15 minutes at room temperature. After three washes with 0.02 % Tween20 in PBS (PBST), cells were blocked for 10 min in 3 % BSA-1xPBS at room temperature and washed again three times in PBST. Cells were incubated for 3h in primary antibodies in 3 % BSA-1xPBS and washed three times with PBST. Secondary antibodies (Thermo Scientific, 1:400 in 3 % BSA-1xPBS) where applied for 1h at room temperature. To stain nuclei, Hoechst33342 (1:10000) was added for 5 min to cells in PBST and finally washed three times. Filipin staining was performed after fixation for 2h at room temperature in PBS (0.05 mg/ml). Primary and secondary antibodies used in this study can be found in the Reagents and Tools Table.

### Fixed-cell microscopy – general acquisition parameters

Immunofluorescently labelled Hela or iNeurons (antibodies indicated in figures and figure legends and details in Reagents and Tools Table) were imaged at room temperature using a Yokogawa CSU-W1 spinning disk confocal on a Nikon Eclipse Ti-E motorized microscope equipped with a Nikon Plan Apochromat 40×/0.40 N.A air-objective lens, Nikon Apochromat 60×/1.42 N.A oil-objective lens and a Plan Apochromat 100×/1.45 N.A oil-objective lens. Signals of 405/488/568/647 fluorophores were excited in sequential manner with a Nikon LUN-F XL solid state laser combiner ([laser line – laser power]: 405 nm - 80 mW, 488 nm - 80 mW, 561nm - 65 mW, 640 nm - 60 mW]) using a Semrock Di01-T405/488/568/647 dichroic mirror. Fluorescence emissions were collected with Chroma ET455/50m [405 nm], 488 Chroma ET525/50m [488 nm], 568 Chroma ET605/52m [561 nm], 633 Chroma ET705/72m [640 nm] filters, respectively (Chroma Technologies). Confocal images were acquired with a Hamamatsu ORCA-Fusion BT CMOS camera (6.5 μm^2^ photodiode, 16-bit) camera and NIS-Elements image acquisition software. Consistent laser intensity and exposure time were applied to all the samples, and brightness and contrast were adjusted equally by applying the same minimum and maximum display values in ImageJ/FiJi.^65^

### Evaluation of Ferritin accumulation in lysosomes

The quantitative measurement of FTH1 accumulation inside the lysosomal mask was performed by seeding HeLa^TMEM192-HA^ control and mutant cell lines into 24-well 1.5 high performance glass bottom plates (Cellvis, 24–1.5H-N) and treated according to the experimental plan. Fed and treated cells were fixed according to the procedure stated above, stained for ferritin (FTH1), lysosomes (HA) and DNA (SPY-DNA555) and imaged using a Nikon Plan Apochromat 40×/0.40 N.A air-objective lens. 12 randomly selected positions (8μm z-stacks) were acquired were acquired using the HCA-module in NIS-Elements. For image analysis, CellProfiler^67^ was used for the quantitative analysis of FTH1 colocalization with the HA-derived lysosomal mask. Plotting of microscopy data was performed in Prism. Primary and secondary antibodies used in this study can be found in the Reagents and Tools Table.

## 3D STRUCTURED-ILLUMINATION-MICROSCOPY

### Fixed cell 3D-SIM sample preparations

Fixed cell 3D-SIM samples were prepared as described.^68^ Briefly, HeLa^TMEM192-HA^ control and mutant cell lines were seeded on 18x18 mm Marienfeld Precision cover glasses thickness No.1.5H (tol. ± 5 μm) and cultured at the indicated conditions / treatments. Cells were fixed at 37°C in 4 % paraformaldehyde (Electron Microscopy Science) for 30 min and permeabilized for 15 min with 0.5 % Triton X-100 in PBS at room temperature. After three washes with 0.02 % Tween20 in PBS (PBST), cells were blocked for 10 min in 3 % BSA-1xPBS at room temperature and washed again three times in PBST. If required, cholesterol molecules were labeled with Filipin for 2h room temperature in 1x PBS (0.1 mg/ml), before washing the sample with PBST twice to remove excess label. Primary antibody incubation was performed over night at 4°C with gentle rocking in 3 % BSA – 1x PBS, followed by three 5 min washes with PBST. Secondary antibody incubation (1:400 in 3 % BSA-1x PBS) was performed at room temperature for 1h with gentle rocking. Samples were washed three times for 5 min in 1xPBST. Before mounting on glass slides, coverslips were washed once in 1xPBS and mounted in Vectashield (Vector Laboratories, H-1000–10). Primary and secondary antibodies used in this study can be found in the Reagents and Tools Table.

### 3D-SIM microscopy – acquisition parameters

3D-SIM microscopy was performed on a DeltaVision OMX v4 using an Olympus 60x / 1.42 Plan Apo oil objective (Olympus, Japan). The instrument is equipped with 405 nm, 445 nm, 488 nm, 514 nm, 568 nm and 642 nm laser lines (all >= 100 mW) and images were recorded on a front-illuminated sCMOS (PCO Photonics, USA) in 95Mhz, 512x512px image size mode, 1x binning, 125 nm z-stepping and with 15 raw images taken per z-plane (5 phase-shifts, 3 angles). Raw image data was computationally reconstructed using CUDA-accelerated 3D-SIM reconstruction code (https://github.com/scopetools/cudasirecon) based on.^69^ Optimal optical transfer function (OTF) was determined via an in-house build software, developed by Talley Lambert from the NIC / CBMF (GitHub: https://github.com/tlambert03/otfsearch, all channels were registered to the 528 nm output channel, Wiener filter: 0.002, background: 90). ChimeraX was used for 3D renderings if imaging data.

### Live-cell 3D-SIM sample preparations

MatTek 35 mm Dish, High Precision 1.5 Coverslip were coated for 2 h with poly-L-lysine a 37°C before washing excess solution off with three 1xPBS washes. Cells were seeded in dishes and cultured / treated as indicated. On day of experiment, cells were incubated with PKmitoRed (1:1000) for one hour at 37°C washed with warm medium to remove excess dye. For assessing fusion-competency of lysosomes in NPC2^−/−^ mutants, cells were seeded in MatTek 35 mm dishes (see above) and loaded with Alex647-conjugated Dextran o/n (1:200 dilution) at 37°C. The next day, cells were stained with LysoTracker Red DND-99 for 1h at 37°C (1:5000), washed twice with PBS and medium replaced with fresh, warm growth medium.

### Live-cell 3D-SIM microscopy – acquisition parameters

3D-SIM microscopy was performed on a DeltaVision OMX v4 using an Olympus 60x / 1.42 Plan Apo oil objective (Olympus, Japan). The instrument is equipped with 405 nm, 445 nm, 488 nm, 514 nm, 568 nm and 642 nm laser lines (all >= 100 mW) and images were recorded on a front-illuminated sCMOS (PCO Photonics, USA) in 286Mhz, 512x512px image size mode, 1x binning, 125 nm z-stepping and with 15 raw images taken per z-plane (5 phase-shifts, 3 angles). ~10 %T laser was used at 5–20 ms exposure times. 0.750 – 1 μm (for mitochondria) or 2 μm (for lysosomes) thick z-stacks were recorded for each timepoint / field of view. Raw image data was computationally reconstructed using CUDA-accelerated 3D-SIM reconstruction code stated above.

## TRANSMISSION ELECTRON MICROSCOPY (TEM)

HeLa^TMEM192-HA^ Control and mutant cells were grown on Aclar plastic coverslips in above stated growth conditions until 70–80 % confluency was reached, washed twice in 1x PBS and fixed with a fixation mixture of 2 % formaldehyde and 2.5 % glutaraldehyde in 0.1 M Sodium Cacodylate buffer, pH 7.4 for 1h at room temperature. Sample preparation and microscopy was performed by the Harvard Medical School Electron microscopy facility (https://electron-microscopy.hms.harvard.edu/methods).

## CRYO-PLASMA FIB - CRYO-ELECTRON TOMOGRAPHY (CRYO-ET)

### Cryo-ET sample preparation and freezing

HeLa^TMEM192-HA^ Control and NPC2^−/−^ cells were cultured on EM grids as follows: 200-mesh gold grids with Silicon Dioxide R1/4 film (Quantifoil) were plasma cleaned, coated by incubation with 1 mg/mL Poly-L-Lysine (Sigma, P2636) solution in 0.1 M Borate Buffer (pH 8.5 in distilled water, autoclaved) for 2h and washed twice with PBS. One day before plunging, a 150 μL drop of ~150 cells/μL was added on top of each grid and placed in a well of a 4-well 35 mm cell culture dish (Greiner bio-one, 627170); after 2h of settling time, DMEM medium was added to a final volume of 2 mL per dish. The next day, cells were starved for 6h in phenol red-free EBSS and 10% glycerol was added to the medium few minutes before plunging. Samples were plunged into ethane/propane with a Vitrobot Mark IV (Thermo Fisher Scientific), with application of 4 μL of EBSS medium and with the following settings: room temperature, humidifier 70 %, blot force 8, blot time 9s. After plunging, the grids were clipped into autogrids with cutout for FIB-milling in a custom clipping station.^70^

### Focused ion beam (FIB) milling

TEM-transparent lamellae were produced in a commercially available Arctis cryo-Plasma Focused Ion Beam (cryo-PFIB) instrument (Thermo Fisher Scientific, Eindhoven, The Netherlands) equipped with a robotic sample delivery device (termed “Autoloader”), compustage, NiCol-scanning electron microscope (SEM) column and Tomahawk-focussed ion beam (FIB) column. Pre-clipped grids were assembled in the standard multispecimen cassette holder so that the cutouts later face the ion beam on the compustage. A Xenon ion beam was used for all described steps. After powering on and aligning the beams in the XT user interface, all consecutive steps were carried out using the proprietary Arctis WebUI software (Version 1.0).^70,71^ The grid template sets the parameters for the initial mapping of the grid from the SEM or FIB as well as for the initial protective coating, and the final sputter fiducials on the polished lamella. We used here a modified version of the “Electron tileset with auto deposition”. First, a tiled overview perpendicular to the grid was acquired with the SEM with a dwell time of 3 μs and a horizontal field width of 256 μm. Then, points of interest (POIs) were placed at suitable cell positions manually. To protect the leading edge of these positions while milling, a three-step protocol of sputter-, chemical vapor-, and sputter deposition was carried out. The sputtering process was executed by milling a calibrated regular pattern into an in-built platinum target with a 12 kV Xenon beam at a current of 70 nA for 120s in order to deposit a thin film of atomic platinum. The platinum fiducials on the end of lamella preparation were induced by the same process but milling only for 5 s. Chemical vapor deposition was executed by heating the attached gas injection system to 28°C and opening the shutter for 50s in order to deposit an organo-metallic layer of trimethyl(methylcyclopentadienyl)-platinum(IV) with a thickness of several μm on the sample.

The lamella template sets the imaging and milling parameters for the retrieval of the POIs in the ion beam, the milling angle search, the ion beam milling, and the final image acquisition. The set final lamella thickness was refined to 120 ± 10 nm depending on the ice thickness of the respective grid. Briefly, the 30 keV Xenon beam milling procedure was as follows: eucentric height and the maximum milling angle of −18° were refined before milling 0.5 μm wide stress relief cuts at a distance of 10 μm to each side of the intended lamella using a 1.0 nA ion beam. Three milling steps were then used to remove material above and below the intended lamella position: (i) rough milling at 3.0 nA to 1 μm thickness, (ii) 0.3 nA to 500 nm, and (iii) 0.1 nA to the 300 nm. The respective Silicon depth correction factors were (i) 0.4, (ii) 0.7, and (iii) 0.88. Afterwards, the lamella was polished at a current of 30 pA to 110–130 nm. In some cases, remnants of the cell top surface with its organometallic layer had to be removed in addition to make the full tilt range in cryo-ET accessible.

### Cryo-ET data acquisition and Processing

TEM data acquisition was performed on a Krios G4 at 300 kV with Selectris X energy filter and Falcon 4i camera (Thermo Fisher Scientific, Eindhoven, The Netherlands) using Tomo5 (Version 5.12.0, Thermo Fisher Scientific). Tilt series were acquired at a nominal magnification of 42,000X (pixel size 2.93 Å) using a dose-symmetric tilt scheme with an angular increment of 2°, a dose of 2 e^−^/Å^2^ per tilt and a target defocus between −3 and −6 μm. Tilt series were collected ranging from −48° to +60° relative to the lamella pretilt., and frames were saved in the EER file format. The positions for tilt series acquisition were determined by visual inspection of 11500X magnification “search” montage maps acquired in thin areas of the sample. For publication display, search maps were cleaned and destriped using the Fiji LisC macro and the contrast was enhanced using Contrast Limited Adaptive Histogram Equalization^72^ (https://github.com/FJBauerlein/LisC_Algorithm, Figure 4C). Tilt series were acquired of lysosome-like structures. Tilt series frames were motion-corrected with Relion’s implementation of Motioncorr2^73^ for EER files.^59^ Alignment and CTF-correction was performed in IMOD^74^ (v.4.10.49, RRID:SCR_003297, https://bio3d.colorado.edu/imod/) and reconstruction by AreTomo^75^ (v.1.3.3) by using an adjusted version of the TomoMAN wrapper scripts (https://doi.org/10.5281/ZENODO.4110737). Tomograms at 2×binning (IMOD bin 4) with a nominal pixel size of 11.72 Å were denoised using cryo-CARE (https://github.com/juglab/cryoCARE_T2T).^76^

### Cryo-ET dataset annotation and analysis

Membrane thickness was measured in Gwyddion^77^ (v.2.63, http://gwyddion.net/) from unbinned, ctf-corrected tomograms after preprocessing with IMOD and EMAN.^78^ To achieve the necessary contrast, tomograms were oriented in the 3Dmod slicer so that the interleaflet space was optimally visible. Tomograms were then rotated, low-pass filtered to the Nyquist frequency (EMAN2, v.2.99.47, https://blake.bcm.edu/emanwiki/EMAN2), and trimmed to the multilamellar bodies’ volumes. Then, tomograms were averaged in z along the slices containing visible membranes and converted to a single 16-bit TIF image. In Gwyddion, the tomogram was inverted and its minimum intensity value shifted to zero. Afterwards, profiles of similar length were extracted along arbitrary lines perpendicular to the membrane. At least four distinct profiles were placed across the whole membrane, each averaging 64 pixels perpendicular to the drawn line. Afterwards, the individual profiles were averaged in OriginLab 2023. Subsequently, peaks were detected and their full width at half maximum (FWHM) and peak-to-peak distance was analyzed automatically. For attenuated peaks, the peak- as well as the FWHM-positions were refined manually. Plots were created in Prism.

### Tomogram segmentation

All membranes in the tomogram were segmented with Membrain-Seg^79^ (https://github.com/teamtomo/membrain-seg) using the publicly available pretrained model (v9), with the exception of the membranes of the multilamellar vesicles (MLV), which were segmented with TomosegmemTV^79,68^ (v.1.0, https://sites.google.com/site/3demimageprocessing/tomosegmemtv) due to their aberrant membrane spacing. All segmentations were then merged and manually refined in Amira.3D (Thermo Fisher Scientific), and final renderings were generated in ChimeraX.^70^

## Supplementary Material

Supplement 1 **Table S1**. Generation of CRISPR-edited cell lines for interrogation of lysosomal storage disease gene function analysis. This file contains gRNA sequences as well as allele sequencing results for all edits examined. Relevant to Figure S1E–G.

Supplement 2 **Table S2**. nMOST proteomic and lipidomic analysis of 33 LSD cell lines (total proteome). Related to Figure 2, S1G,H and S2.

Supplement 3 **Table S3**. nMOST Cross-Ome analysis of 33 LSD cell lines (whole cell). Related to Figure 2, S1G,H and S2.

Supplement 4 **Table S4**. nMOST proteomic and lipidomic analysis of Control, *LIPA*^−/−^, *GAA*^−/−^, *NPC1*^−/−^ and *NPC2*^−/−^ HeLa^TMEM192-HA^ cells under fed and starved (EBSS) conditions. Related to Figure 3, S3A,B, 5A,B,E–G, S5C–H.

Supplement 5 **Table S5**. TMT-based proteomic analysis of Control and *NPC2*^−/−^ HeLa^TMEM192-HA^ with and without FAC addition. Relevant to Figure 6D–G, S6F–K, Figure 7 and S7.

Supplement 6

## Data Availability

Proteomic data and analysis files part of this study are deposited at ProteomeXchange Consortium by the PRIDE partner.^80^ The PRIDE project identification number is PXD049336 (TMTpro experiments). Raw files collected by nMOST are available on MassIVE, assession number: MSV000094201. Macros and pipelines used in this work can be found on GitHub.
