# Supplementary material for: Lysosomal storage disease proteo/lipidomic profiling using nMOST links ferritinophagy with mitochondrial iron deficiencies in cells lacking NPC2
Source: bioRxiv. 2024 Mar 27:2024.03.26.586828. Preprint. [Version 1] doi: 10.1101/2024.03.26.586828 (PMC10996675; doi:10.1101/2024.03.26.586828)
Supplement: Supplementary file 1 [file NIHPP2024.03.26.586828v1-supplement-6.pdf]

bioRxiv preprint doi: https://doi.org/10.1101/2024.03.26.586828; this version posted March 27, 2024. The copyright holder for this preprint (which was not certified by peer review) is the author/funder, who has granted bioRxiv a license to display the preprint in perpetuity. It is made available under aCC-BY 4.0 International license.

SUPPLEMENTAL FIGURES

Supplemental Figure 1

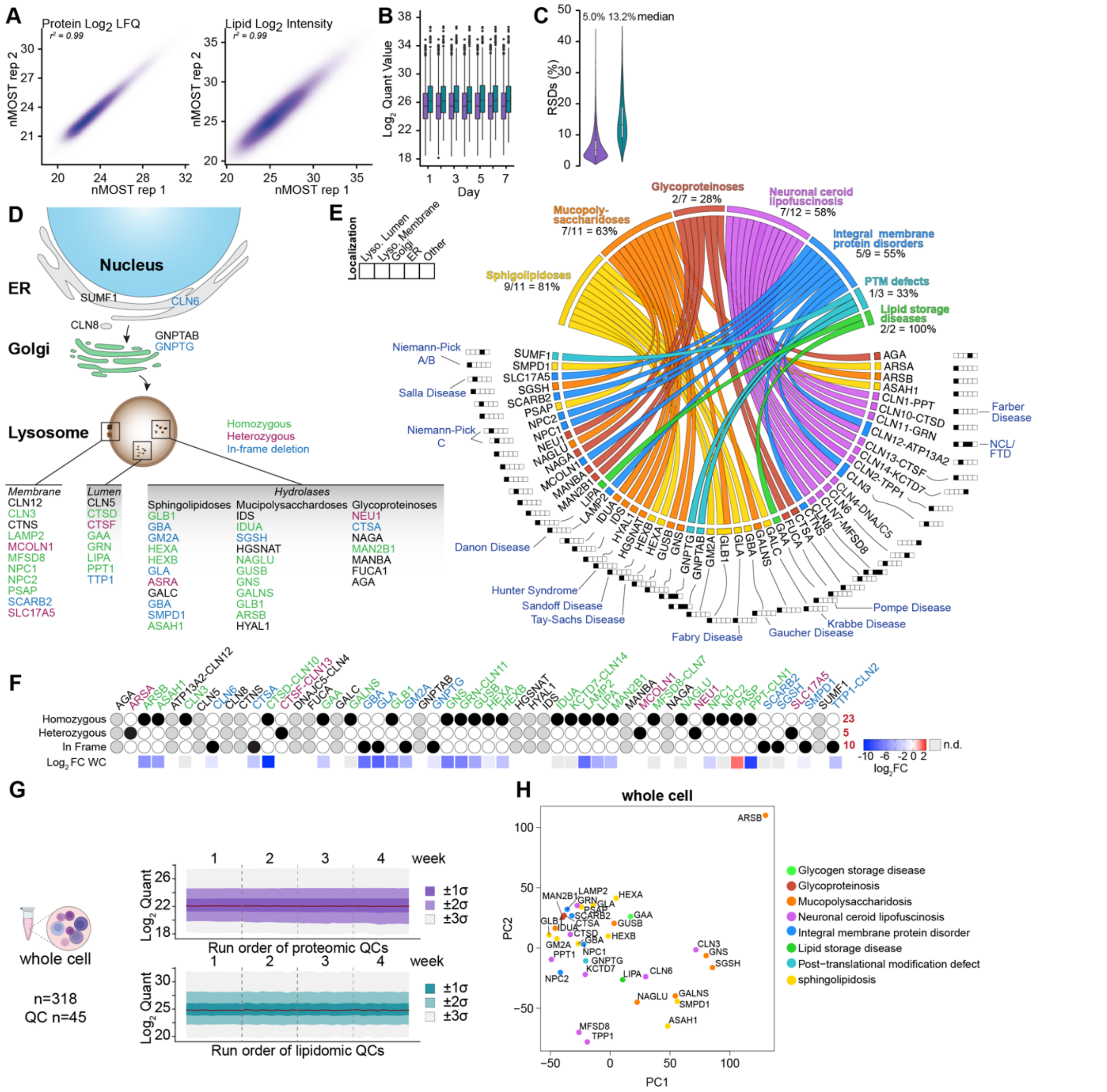

Figure S1: Benchmarking of nMOST and application to cells lacking LSD proteins.
(A) Correlation of $\log_2$ label-free quantification (LFQ) protein (left panel) and lipid biomolecules (right) of two nMOST runs. **(B)** Quantification of number of $\log_2$ quant value (protein and lipid) over a 7-day acquisition period using nMOST. **(C)** Violin plot depicting % relative standard deviations (RSDs) for both quantified protein and lipid identifications. **(D)** Schematic summarizing 52 LSD proteins and their localization properties when known. **(E)** Diagram showing how individual LSD genes map on disease classifications. Common names associated with individual LSD genes are indicated, as is their localization based on compartments shown in the inset. **(F)** Summary of gene editing champaign with the goal of creating mutants across LSD genes in HeLa$^{TMEM192-HA}$ cells. Black circles indicate the status of mutants obtained. Gray circles indicate no clones for the indicated genes. Lower panel shows $\log_2$ FC for all detected LSD proteins in either whole cell extracts or Lyso-IPs from the indicated mutant cell line based on nMOST. **(G)** Log_2 LFQ for total proteomes/lipidomes (left panel) for 363 total samples analyzed over a 4-week data collection session (318 whole cell extracts with each LSD mutant, WT HeLa$^{TMEM192-HA}$ control cells all in quadruplicate biological replicates, and 45 MS QC samples). **(H)** PCA blots for combined proteome and lipidome across the LSD mutants in this study.

bioRxiv preprint doi: https://doi.org/10.1101/2024.03.26.586828; this version posted March 27, 2024. The copyright holder for this preprint (which was not certified by peer review) is the author/funder, who has granted bioRxiv a license to display the preprint in perpetuity. It is made available under aCC-BY 4.0 International license.

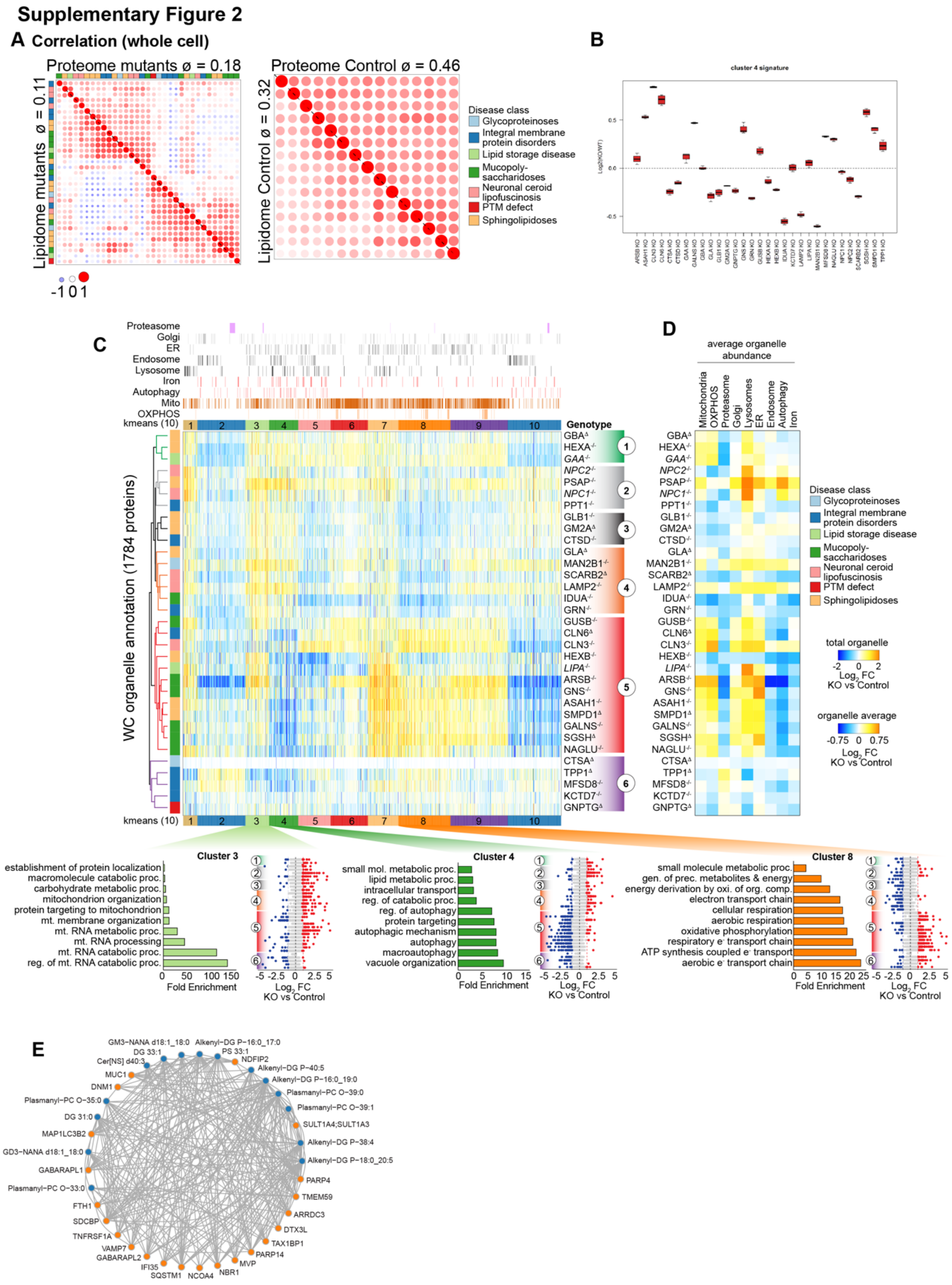

Figure S2. Analysis of cells lacking LSD proteins using nMOST.
(A) Heatmap depicting correlation of proteome and lipidomes for LSD mutants (top) and controls (bottom). **(B)** Summed cross-correlation plot for cluster 4 across the LSD mutant cells plotted as $\log_2$FC (KO/WT). **(C)** Heatmap of proteome for $\log_2$FC [Mutant / Control] of annotated organelle proteins across LSD mutants. Fold enrichment of GO terms associated with proteins in three select cluster (#3,4,8) are plotted below. Individual protein abundance ($\log_2$FC [Mutant / Control; blue > -1, red < 1) for each cluster is plotted besides the GO term analysis. **(D)** Heatmap ($\log_2$FC [Mutant / Control]) of average proteome abundance across the indicated organelle of LSD mutants. **(E)** Correlated enrichment of LysoPC species with autophagy receptors.

bioRxiv preprint doi: https://doi.org/10.1101/2024.03.26.586828; this version posted March 27, 2024. The copyright holder for this preprint (which was not certified by peer review) is the author/funder, who has granted bioRxiv a license to display the preprint in perpetuity. It is made available under aCC-BY 4.0 International license.

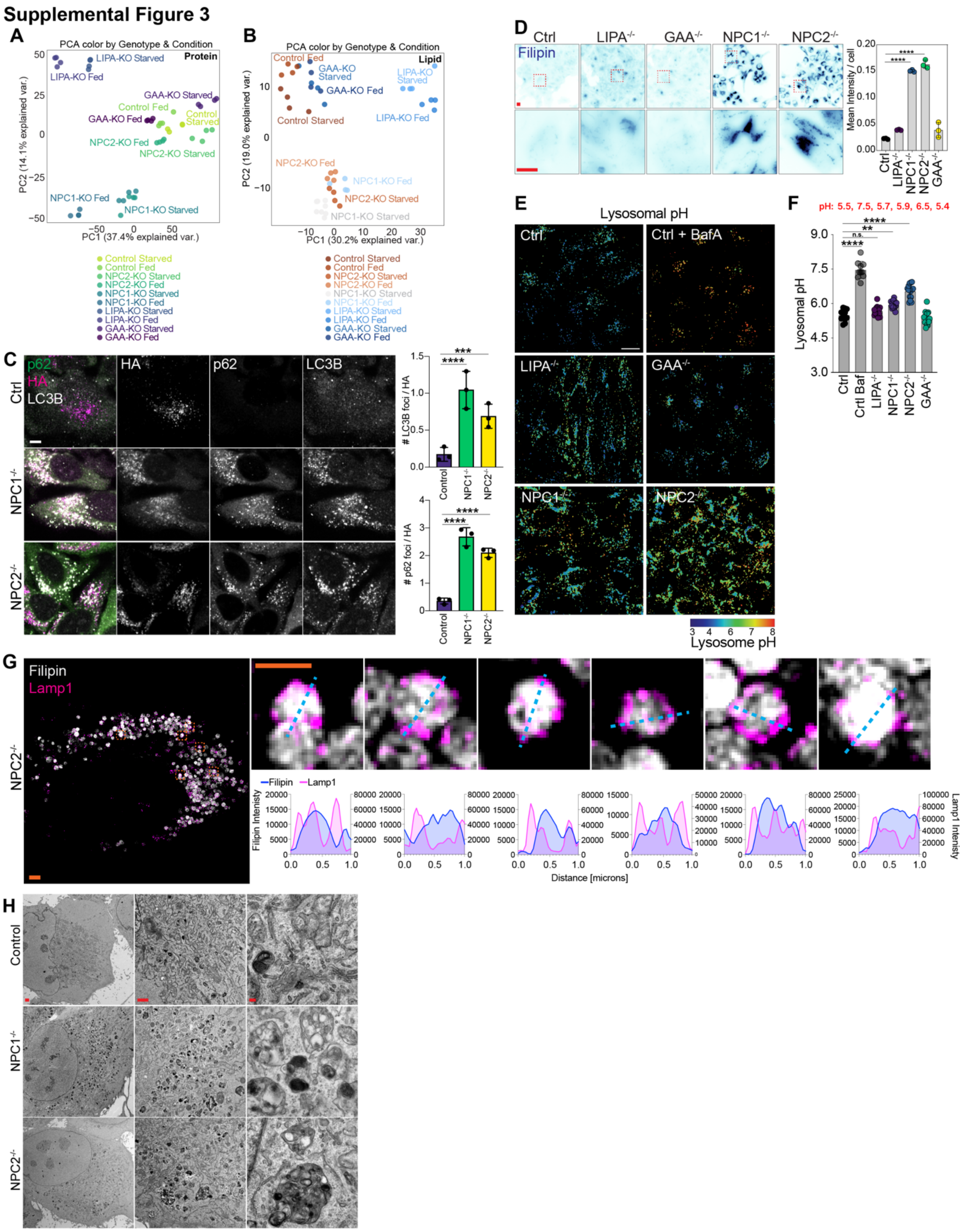

Figure S3. Functional analysis of lysosomes in cells lacking LIPA, GAA, NPC1 or NPC2.
(A,B) PCA analysis of 4KO proteomic (panel A) and lipidomic (panel B) data from nMOST analysis of the indicated cell lines under Fed or EBSS (6 h) conditions. Data based on quadruplicate biological replicate nMOST measurements. **(C)** Confocal imaging of Control, *NPC1*$^{-/-}$ and *NPC2*$^{-/-}$ cells immunostained with a-LC3, a-SQSTM1, and a-HA to detect TMEM192^{HA}. Scale bar = 5 μm. Quantification was performed on three biological replicates with 5 stacks in each replicate. MAPLC3B: p(****) <0.0001; p(***) = 0.0002; p(*)=0.0129 & 0.0157. p62/SQSTM1: p(****) <0.0001. Data from quadruplicate replicates, ordinary one-way ANOVA with multiple comparisons, alpha = 0.05. Error bars depict S.D. **(D)** Wide-field fluorescent images of Hela control and 4KO cell lines stained for cholesterol with Filipin. Quantification of mean Filipin intensity per cell is plotted below (data from three biological replicates, 20 image stacks per repeat; genotype(N)): Ctrl(1424), *LIPA*$^{-/-}$(2123), *GAA*$^{-/-}$ (1791), *NPC1*$^{-/-}$ (1773), *NPC2*$^{-/-}$ (3270)). p(****) <0.0001, ordinary two-way ANOVA with multiple comparisons, alpha = 0.05; error bars depict S.D. Scale bar = 20 μm. **(E,F)** pH measurements for 4KO cells using confocal imaging (panel E) and ratiometric analysis to determine the pH (panel F) was performed as described in **METHODS**. Scale bar =

bioRxiv preprint doi: https://doi.org/10.1101/2024.03.26.586828; this version posted March 27, 2024. The copyright holder for this preprint (which was not certified by peer review) is the author/funder, who has granted bioRxiv a license to display the preprint in perpetuity. It is made available under aCC-BY 4.0 International license.

20 µm. Each data point represents one field of view; Repeats per genotype (N): Ctrl(12), Ctrl + BafA(10), *LIPA*$^{-/-}$, *GAA*$^{-/-}$, *NPC1*$^{-/-}$, *NPC2*$^{-/-}$(15). p(****) <0.0001; p(**) = 0.0022; ordinary one-way ANOVA with multiple comparisons, alpha = 0.05; error bars depict S.D. **(G)** Line trace plots for individual Filipin-positive lysosomes and a-LAMP1 signal derived from 3D-SIM imaging. Scale bar = 2 µm and 1 µm (inset). **(H)** Control, *NPC1*$^{-/-}$ or *NPC2*$^{-/-}$ cells were examined by transmission electron microscopy. Scale bars = 1 µm (left & middle panel) and 100 nm (right panel).

Supplementary Figure 4

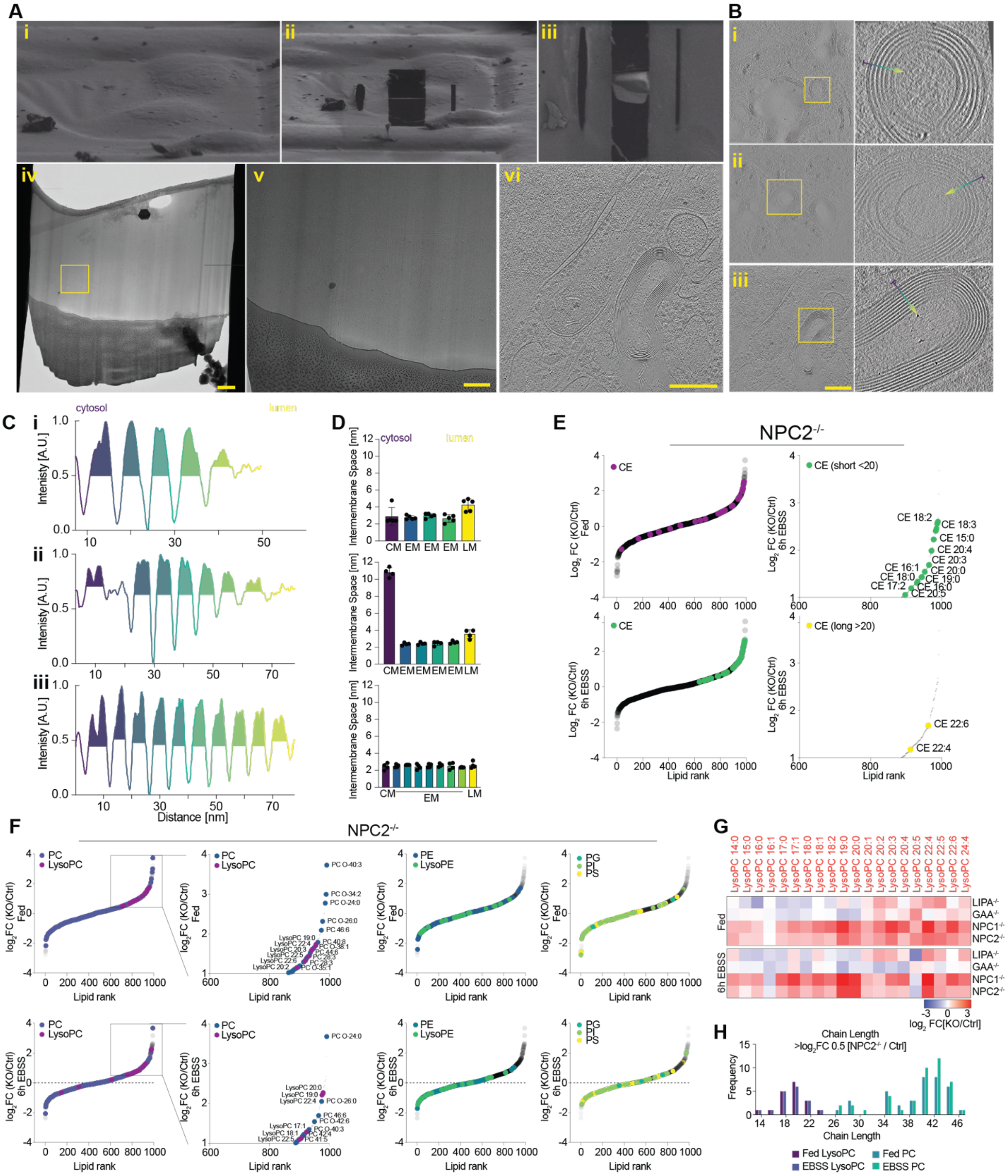

Figure S4. Visualization of multi-lamellar membranes in *NPC2*$^{-/-}$ lysosomes by cryo-ET.
(A) Example images of the cryo-PFIB and cryo-ET workflow. Vitrified cells before (i) and after (ii) lamella preparation by cryo-PFIB milling. (iii) final SEM view of milled and polished lamella. (iv-v) Lamella overview with zoom-in on a MLV containing area. (vi) Reconstructed tomogram showing an MLV. Scale: (i-ii) 156 µm horizontal field width (HFW), (iii) 124 µm HFW. Scale bar = (iv) 1 µm (v) 500 nm (vi) 250 nm. **(B)** Overview images and zoom-ins of three tomograms depicting MLV membrane stacks. Scale bar = 250 nm. **(C)** Averaged, inverted intensity along the arrows from B to determine membrane thickness. The gradient indicates the measurement direction from cytosol (purple) to lumen (yellow). Membrane peaks are colored to indicate their full width at half maximum. **(D)** Intermembrane Space of selected MLVs between adjacent membrane pairs. The gradient indicates

bioRxiv preprint doi: https://doi.org/10.1101/2024.03.26.586828; this version posted March 27, 2024. The copyright holder for this preprint (which was not certified by peer review) is the author/funder, who has granted bioRxiv a license to display the preprint in perpetuity. It is made available under aCC-BY 4.0 International license.

the measurement direction from cytosol (purple) to lumen (yellow). **(E)** Ranked lipid $\log_2$FC abundance of *NPC2*$^{-/-}$ lipidome for fed and 6h EBSS nutrient starvation conditions. Cholesterol esters (CE) are highlighted in color on top of the overall lipidome spread. The lower row depict ranking of short or longer CE in 6h EBSS nutrient starvation conditions against the whole lipidome rank. Data based on quadruplicate biological replicate nMOST measurements. **(F)** Ranked lipid $\log_2$FC abundance of phospholipids in *NPC2*$^{-/-}$ lipidome for fed and 6h EBSS nutrient starvation conditions. Lysosomal specific phospholipids (LysoPC/LysoPE) are highlighted in color on top of the overall lipidome and parent-lipid class spread. Lower row depicts the lipidome of annotated lipids in 6h EBSS nutrient starvation conditions against the whole lipidome rank. Data based on quadruplicate biological replicate nMOST measurements. **(G)** Heatmap depicting $\log_2$FC of LysoPC species in either Fed and EBSS-treated Control and 4KO cells. Data based on quadruplicate biological replicate nMOST measurements. **(H)** Histogram depicting frequency of (Lyso-)PCs enriched $\geq$ 0.5 $\log_2$FC in [*NPC2*$^{-/-}$/Control] against their chain length. Data based on quadruplicate biological replicate nMOST measurements.

bioRxiv preprint doi: https://doi.org/10.1101/2024.03.26.586828; this version posted March 27, 2024. The copyright holder for this preprint (which was not certified by peer review) is the author/funder, who has granted bioRxiv a license to display the preprint in perpetuity. It is made available under aCC-BY 4.0 International license.

Supplementary Figure 5

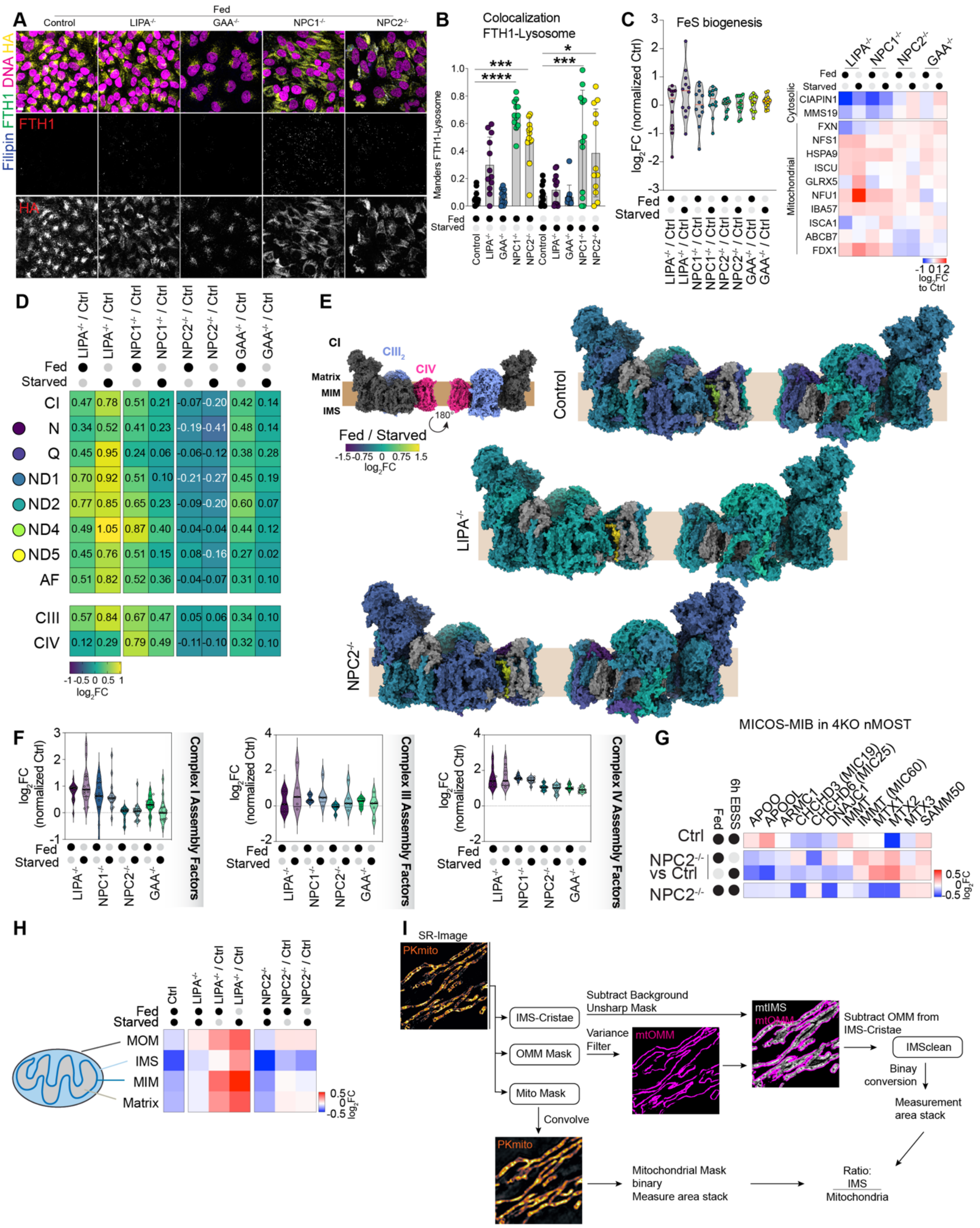

Figure S5: Ferritin accumulation and loss of mitochondrial cristae/OXPHOS systems in *NPC2*$^{-/-}$ cells.

(A) Confocal fluorescent images of control and 4KO cells in Fed cells immunostained with α-FTH1 and α -HA to detect TMEM192^{HA}. Cholesterol-rich lysosomes were stained with Filipin and nuclei were stained with DNA SPY555. Scale bar = 20 µm. **(B)** Images from panel A and an analogous experiment with EBSS treatment (6 h) were quantified by measuring Mander's overlap between FTH1 signal and the lysosome mask provided by a-HA staining. Data from 12 image stack per condition; genotype (Number of cells Fed/EBSS): Ctrl(3307/1973), *LIPA*$^{-/-}$ (1996/1137), *GAA*$^{-/-}$(1401/956), *NPC1*$^{-/-}$(1211/2104), NPC2$^{-/-}$(1629/749). Error bars depict S.D. **(C)** Log$_2$FC heatmap for components of the mitochondrial and cytosolic FeS cluster biogenesis system for 4KO cells in Fed and EBSS-treated cells based on quadruplicate replicate nMOST data. **(D)** Log$_2$FC values for modules within OXPHOS for 4KO cells in Fed and EBSS-treated cells based on quadruplicate replicate nMOST data. **(E)** Log$_2$FC of replisome sub-module abundance comparing Fed versus EBSS treated Control, *LIPA*$^{-/-}$, *GAA*$^{-/-}$, *NPC1*$^{-/-}$, and *NPC2*$^{-/-}$ cells based on quadruplicate biological replicate nMOST data. Legend shows color panel for log$_2$FC values. **(F)** Violin

bioRxiv preprint doi: https://doi.org/10.1101/2024.03.26.586828; this version posted March 27, 2024. The copyright holder for this preprint (which was not certified by peer review) is the author/funder, who has granted bioRxiv a license to display the preprint in perpetuity. It is made available under aCC-BY 4.0 International license.

plots depicting log_2FC values for CI assembly factors (left panel), CIII assembly factors (middle panel) and CIV assembly factors (right panel) for 4KO cells [normalized with Control]. Data based on quadruplicate biological replicate nMOST measurements. **(G)** Heatmap for components of the MICOS-MIB complex in for 4KO cells [normalized with Control]. Data based on quadruplicate biological replicate nMOST measurements. **(H)** Heatmap depicting log_2FC of components of different mitochondrial compartments in either Fed and EBSS-treated Control and 4KO cells. Data based on quadruplicate biological replicate nMOST measurements. (**I**) Schematic summarizing the method used to determine cristae spacing within mitochondria.

Supplementary Figure 6

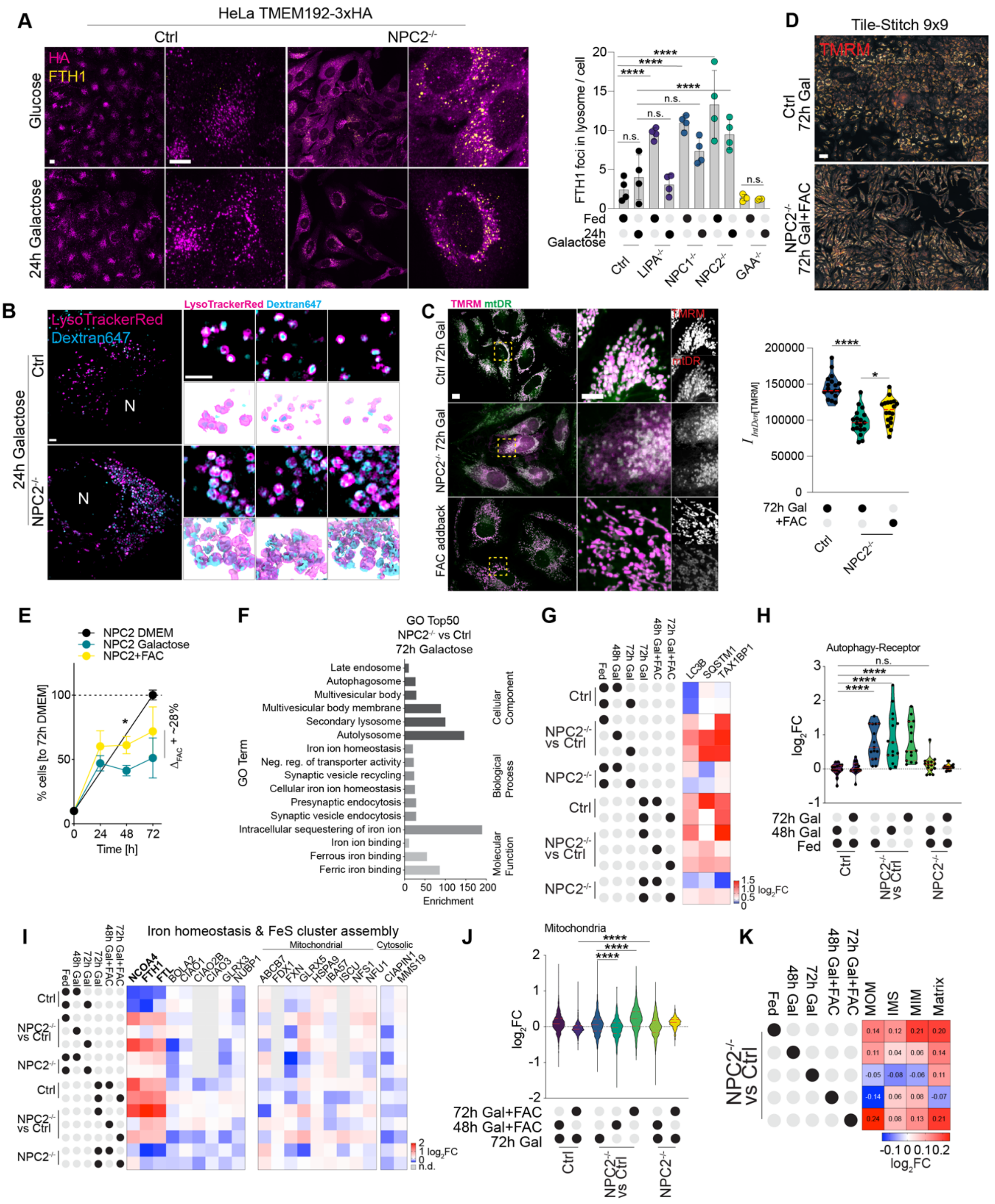

Figure S6. Rescue of mitochondrial cristae in *NPC2*$^{-/-}$ cells by extracellular iron.
(A) Confocal images of Control and *NPC2*$^{-/-}$ cells grown in glucose (Fed) or Galactose (24h) followed by immunostaining with a-HA to detect TMEM192^{HA} and a-FTH1 to detect Ferritin. Scale bar = 10 µm. Right panel

bioRxiv preprint doi: https://doi.org/10.1101/2024.03.26.586828; this version posted March 27, 2024. The copyright holder for this preprint (which was not certified by peer review) is the author/funder, who has granted bioRxiv a license to display the preprint in perpetuity. It is made available under aCC-BY 4.0 International license.

displayed quantification of FTH1 signal overlapping with lysosome staining/cell. Data from biological quadruplicates per sample (each replicate containing 5-9 stacks); p(****) <0.0001, ordinary two-way ANOVA with multiple comparisons, alpha = 0.05. Error bars depict S.D. **(B)** Control or *NPC2*$^{-/-}$ cells grown in Galactose (24h) were treated with dextran conjugated with alexa-647 dye and imaged by structured illumination microscopy. Images derived from 3D-SIM reconstructions are shown. Scale bar 2 µm. **(C)** Stills of spinning-disk live-cell microscopy of Control and *NPC2*$^{-/-}$ cells cultured on Galactose for 72h or on Galactose for 72h with FAC. Mitochondria are stained with TMRM (ΔΨm) and MitoTrackerDeepRed. Scale bar = 10 µm & 5 µm (insets). Right panel shows quantification of $I_{[\mathrm{mtDR\text{-}TMRM}]}$ for indicated genotypes and treatments. Data from four biological replicates (18 stacks per replicates); p(****) < 0.0001, p(*) = 0.0150; unpaired t.test. **(D)** 9x9 tile-stitched overview images of live-cell images of Control and *NPC2*$^{-/-}$ cells in the indicated culture conditions. Mitochondrial membrane potential is visualized by TMRM. Scale bar = 50 µm. **(E)** % of cell count of *NPC2*$^{-/-}$ [relative to growth in 72h DMEM] in Galactose ± FAC over a 72h growth period. **(F)** Top GO terms from the total proteome TMT experiment outlined in **Figure 6D**. **(G,H)** Heatmap (panel F) and violin plot (panel G) for $\log_2$FC [*NPC2*$^{-/-}$/Control] of autophagy receptors in cells cultured in Galactose in the presence or absence of FAC (see **Figure 6D**). p(****) < 0.0001; data based on biological triplicate TMTpro measurements. **(I)** Heatmap of $\log_2$FC [*NPC2*$^{-/-}$/Control] for components of the cytosolic and mitochondrial FeS cluster assembly system as well as the Ferritin system with or without FAC with cells grown in Galactose (see **Figure 6D**). Data based on biological triplicate TMTpro measurements. **(J)** Violin plots for total mitochondrial proteins in Control versus *NPC2*$^{-/-}$ cells grown in Galactose with or without FAC (see **Figure 6D**). p(****) < 0.0001; data based on biological triplicate TMTpro measurements. **(K)** Heatmap depicting $\log_2$ FC of components of different mitochondrial compartments in cells cultured in Glucose and Galactose in the presence or absence of FAC. Data based on biological triplicate TMTpro measurements.

Supplementary Figure 7

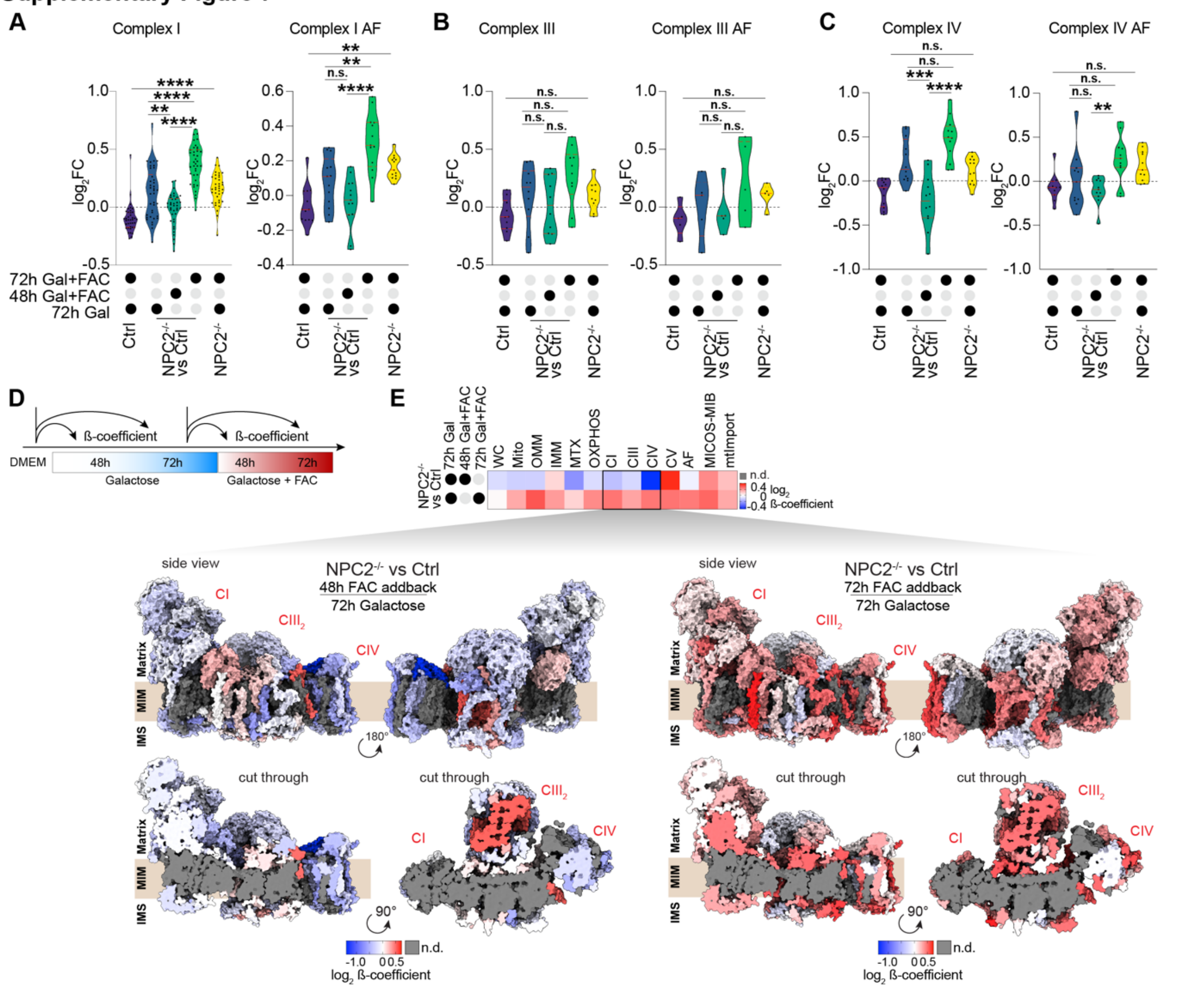

Figure S7. Rescue of OXPHOS complex abundance in *NPC2*$^{-/-}$ cells by extracellular iron.
(A-C) Violin plots of $\log_2$FC values for CI (panel A), CIII (panel B), and CIV (panel C) subunits (left panels) and associated assembly factors (right panels) in *NPC2*$^{-/-}$ versus Control cells grown in Galactose with or without FAC. Complex I: p(****) < 0.0001, p(**) = 0.0036; Complex I AF: p(****) < 0.0001, p(**) = 0.0018; Complex IV: p(****) < 0.0001, p(***) = 0.0002; Complex IV AF: p(**) = 0.0056; ordinary one-way ANOVA with multiple comparisons, alpha = 0.05; data based on biological triplicate TMTpro measurements. **(D)** Schematic of β-coefficient comparisons. **(E)** Log_2FC of β-coefficient of mitochondrial components (see middle heatmap) in Galactose and

bioRxiv preprint doi: https://doi.org/10.1101/2024.03.26.586828; this version posted March 27, 2024. The copyright holder for this preprint (which was not certified by peer review) is the author/funder, who has granted bioRxiv a license to display the preprint in perpetuity. It is made available under aCC-BY 4.0 International license.

either 48h or 72h FAC addback for *NPC2*$^{-/-}$ versus Control cells. Abundance of supercomplex subunits is mapped onto the structure (PDB: 5XTH). Vertical and horizontal cut throughs of the structure are depicted in the lower panels. Legend shows color panel for log_2FC values. Data based on biological triplicate TMTpro measurements.

bioRxiv preprint doi: https://doi.org/10.1101/2024.03.26.586828; this version posted March 27, 2024. The copyright holder for this preprint (which was not certified by peer review) is the author/funder, who has granted bioRxiv a license to display the preprint in perpetuity. It is made available under aCC-BY 4.0 International license.

SUPPLEMENTARY TABLES

Table S1. Generation of CRISPR-edited cell lines for interrogation of lysosomal storage disease gene function analysis. This file contains gRNA sequences as well as allele sequencing results for all edits examined. Relevant to **Figure S1E-G**.

Table S2. nMOST proteomic and lipidomic analysis of 33 LSD cell lines (total proteome). Related to **Figure 2, S1G,H** and **S2.**

Table S3. nMOST Cross-Ome analysis of 33 LSD cell lines (whole cell). Related to **Figure 2, S1G,H** and **S2.**

Table S4. nMOST proteomic and lipidomic analysis of Control, *LIPA*$^{-/-}$, *GAA*$^{-/-}$, *NPC1*$^{-/-}$ and *NPC2*$^{-/-}$ HeLa$^{TMEM192\text{-}HA}$ cells under fed and starved (EBSS) conditions. Related to **Figure 3, S3A,B, 5A,B,E-G, S5C-H**.

Table S5. TMT-based proteomic analysis of Control and *NPC2*$^{-/-}$ HeLa$^{TMEM192\text{-}HA}$ with and without FAC addition. Relevant to **Figure 6D-G, S6F-K, Figure 7** and **S7**.
